# Automated blood glucose regulation for nonlinear model of type-1 diabetic patient under uncertainties: GWOCS type-2 fuzzy approach

**DOI:** 10.1007/s13534-023-00318-3

**Published:** 2023-10-01

**Authors:** Mohanad Elhoushy, Belal A. Zalam, Amged Sayed, Essam Nabil

**Affiliations:** 1 Department of Industrial Electronics and Control Engineering, Faculty of Electronic Engineering, Menoufia University, Menouf, Egypt; 2 Department of Electrical Energy Engineering, College of Engineering and Technology, Arab Academy for Science, Technology and Maritime Transport, Smart Village Campus, Giza, Egypt

**Keywords:** Interval type-2 fuzzy set, Footprint of uncertainty, Grey wolf optimizer, Cuckoo search, Blood glucose level, Uncertainty, Extended Bergman minimal model

## Abstract

Regulating blood glucose level (BGL) for type-1 diabetic patient (T1DP) accurately is very important issue, an uncontrolled BGL outside the standard safe range between 70 and 180 mg/dl results in dire consequences for health and can significantly increase the chance of death. So the purpose of this study is to design an optimized controller that infuses appropriate amounts of exogenous insulin into the blood stream of T1DP proportional to the amount of obtained glucose from food. The nonlinear extended Bergman minimal model is used to present glucose-insulin physiological system, an interval type-2 fuzzy logic controller (IT2FLC) is utilized to infuse the proper amount of exogenous insulin. Superiority of IT2FLC in minimizing the effect of uncertainties in the system depends primarily on the best choice of footprint of uncertainty (FOU) of IT2FLC. So a comparison includes four different optimization methods for tuning FOU including hybrid grey wolf optimizer-cuckoo search (GWOCS) and fuzzy logic controller (FLC) method is constructed to select the best controller approach. The effectiveness of the proposed controller was evaluated under six different scenarios of T1DP using Matlab/Simulink platform. A 24-h scenario close to real for 100 virtual T1DPs subjected to parametric uncertainty, uncertain meal disturbance and random initial condition showed that IT2FLC accurately regulate BGL for all T1DPs within the standard safe range. The results indicated that IT2FLC using GWOCS can prevent side effect of treatment with blood-sugar-lowering medication. Also stability analysis for the system indicated that the system operates within the stability region of nonlinear system.

## Introduction

The number of the people suffering from diabetes mellitus (DM) is increasing yearly. In 2014 the number of patients with DM reached 422 million compared to 108 million in 1980 according to the most recent global report on diabetes by world health organization (WHO) [1], also this number is increased to 537 million in 2021 according to latest atlas edition by international diabetes federation (IDF) [2]. When this disease is left uncontrolled it has dire consequences for health and quality of life, in 2012 diabetes caused 1.5 million deaths, also the higher BGL than the optimal value caused an additional 2.2 million deaths by increasing the risk of cardiovascular and other diseases [1]. Uncontrolled DM leads to different complications include cardiovascular disease, heart attack, instant coma, kidney failure, leg amputation, and others [1, 2]. DM is generally categorized into two types: type-1 and type-2, type-1 DM is a metabolic disease occurs due to autoimmune destruction of *β* cells of pancreas, as a result the pancreas becomes unable to produce enough or any amount of insulin to lower BGL in blood stream, this type is also known as insulin-dependent as patients of this type always need infusing exogenous insulin to survive [3]. While in type-2 DM insulin production by *β* cells is unaffected but patients of this type suffer from insulin deficiency and insulin resistance that the cells of the body can't utilize insulin to regulate BGL [3].

Early detection and future prediction of diabetes can save the patients from many complications and greatly reduces the chance of death thanks to machine learning techniques, because the patient will be able to receive appropriate treatment at an early stage of the disease. In this regard many efforts have been made, in [4–6] different machine learning algorithms were applied to predict and improve the medical diagnosis of DM.

For a healthy person the standard-safe range of BGL is between 70 and 180 mg/dl [7], consequently the main target for patients with DM is to regulate their BGL within the standard-safe range. There are actually many vital organs that contribute to the regulation of BGL such as liver, brain, intestine, pancreas, etc., however the pancreas is the organ that plays a major and influential role in regulating BGL [8]. Pancreas produces and secretes a two very important things, pancreatic hormones and digestive enzymes [8]. These pancreatic hormones are primarily responsible for regulating BGL, it is produced from endocrine cells that called islets of Langerhans [8]. There are different types of pancreatic hormones that are produced and secreted by five types of cells, *β*, *α*, *γ*, *δ*, and *ε* cells that make up the islet of Langerhans. Insulin, amylin, and C-peptide hormones are produced by *β* cells, glucagon hormone is produced by *α* cells, polypeptide hormone is produced by *γ* cells, somatostatin hormone is produced by *δ* cells, and ghrelin hormone is produced by *ε* cells [8]. Among all these hormones which are responsible for regulating BGL insulin and glucagon play an essential role in this regard, where after a meal is digested a proper amount of insulin is secreted into blood by *β* cell of pancreas proportionally to the amount of glucose appears into blood, this amount of insulin stimulates different cells of the body to absorb glucose and use it to produce energy thus insulin lowers BGL. While glucagon is secreted into blood by *α* cell of pancreas when the BGL is at its lowest. Glucagon when is secreted stimulates the liver to produce stored hepatic glucose thus glucagon increases BGL. Based on the above maintaining BGL within the standard-safe range depends essentially on the interplays between insulin and Glucagon [8].

If type-1 DM when is left uncontrolled it leads to a situation called hyperglycemia in which BGL reaches more than 180 mg/dl, this is in turn leads to long term complications like cardiovascular disease, heart attack, blindness, kidney failure, leg amputation, and others [9]. While infusing excessive amount of exogenous insulin leads to a situation called hypoglycemia in which BGL reaches less than 50 mg/dl, this in turn leads to instant coma and death [9].

**Fig. 1:**
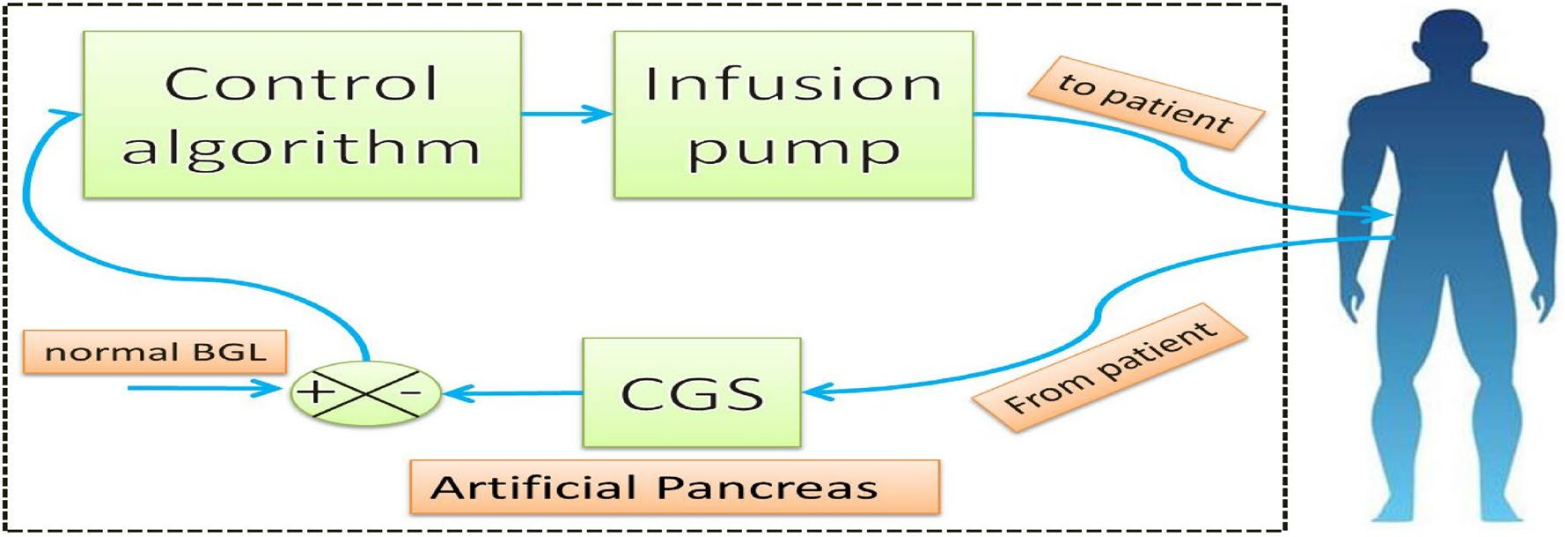
Closed loop control system of AP

Since the discovery of insulin T1DPs have been receiving exogenous insulin manually based on measuring BGL many times during the day and after meals, regardless of the fact that this method is time consuming, unreliable, and painful, it is not suitable at night during sleep hours [10]. Artificial pancreas (AP) has been developed to overcome the defects of the manual method [11]. It is a closed loop control system shown in Fig. 1 in which insulin is infused automatically by infusion pump according to both the readings of a continuous glucose sensor (CGS) and the control algorithm that calculates the optimal rate of exogenous insulin infusion [10].

There is required a secure and reliable communication technology, recently several methods have been developed to improve medical equipment, whale optimized weighted fuzzy-based cluster head selection algorithm was used in [12] to improve communication performance for IoMT-based systems on the 5G network.

Closed loop AP is primarily depends on the mathematical model of T1DP, unfortunately it faces a great challenges when it is applied in practice to a real T1DP because of two reasons: the first reason from the patient side like parametric uncertainty (the parameters of the model vary from patient to other as well as the parameters vary within the same patient), uncertain meal disturbance, and physical exercise that can burn glucose, thus the dose of insulin needs to be continually changed [13]. And the second reason is related to uncertainties that affect the whole control system in general.

Since designing a robust controller depends primarily on a mathematical model, over time many mathematical models have been developed to describe glucose-insulin physiological system for T1DP [14]. It is classified into two types intravenous and subcutaneous based on the way of the measurements of insulin and glucose [14]. Because it is less complex than the others and keeps the minimal state variables necessary to describe the relationship between glucose and insulin for diabetic patient, an intravenous glucose tolerance test (IVGTT) of the Bergman minimal (BM) model [15] is regarded as the most popular in research on artificial pancreas. The three differential equations with its states will model the complete behavior of glucose and insulin kinetics for patients. The first one indicates BGL, where the other one reveals the remote insulin, and the last equation indicates plasma insulin level. By doing so, the BM model helps academics overcome one of their biggest challenges when working with several nonlinear differential equations.

For reasons of high cost and reliability measuring all state variables practically is not possible, so the control algorithm of AP should rely only on the readings of the CGS [14].

So the motivation behind conducting this study is to provide simple-accurate and robust closed loop controller to regulate BGL for T1DP within the standard safe range between 70 and 180 mg/dl which utilizes only the readings of the CGS since the measurement of remote insulin and plasma insulin level is practically not possible, it is also should take into account the aforementioned challenges that T1DP always faces during the infusion of exogenous insulin. After food has been digested as the BGL for T1DP rises above the basal glucose level, the output of the controller must infuse an appropriate amount of exogenous insulin into the bloodstream of T1DP to avoid hyperglycemia situation. The insulin infusion rate must also be closely proportional to the amount of glucose obtained from food because an excessive infusion of exogenous insulin leads to a hypoglycemia situation. Moreover, the output signal of the controller must restrict to be positive or non-negative since insulin infusion rate practically is always non-negative, consequently no excessive controller or algorithm are needed to infuse glucagon or glucose to avoid hypoglycemia and this also enhances the simplicity of the design and reduces complexity and cost.

### Related work

Recently, the control algorithm of AP has been proposed in the literature based on linear control approach and nonlinear control approach. In [16–18] PID Controller was designed to regulate BGL of T1DP based on continuous glucose measurements but the integral action may increase the insulin overdose and hence increasing the chance of hypoglycemia, an improved PID algorithm based on switching off PID controller prior to meal bolus and then restarting it according to switching strategy was proposed in [19], PID controller with insulin feedback algorithm to prevent excessive intake of exogenous insulin was proposed in [14, 20], optimal *H*^∞^ switching controller based on a group of basic insulin infusion rates and a rule that switches the insulin infusion rate from one value to another was proposed in [21], in [22] linear parameters varying model was utilized to consider a time-varying characteristics of the problem dynamics at the control law. Since the physiological system of glucose-insulin is nonlinear and its parameters are uncertain vary from patient to other, it is preferable to use with nonlinear systems a nonlinear control approach rather than a linear control approach to increase stability and performance of the system. In [23, 24] sliding mode controller is designed to regulate BGL of T1DP, also in [25] terminal synergetic and state feedback linearization based controllers were proposed, a backstepping controller was designed in [13], adaptive backstepping controller was designed in [26]. For mentioned control approaches in [13, 23–26] it provides satisfactory performance in regulating BGL of T1DPs against parametric uncertainty and meal disturbance however it considered in the design the possibility of measuring all state variables which practically is not true for reasons of high cost and reliability. In [27–29] model predictive control (MPC) was utilized to regulate BGL of T1DP, it has the ability to deal with parametric uncertainty of glucose-insulin physiological system however the computational of iterative online optimization process is complex and its efficiency depends on how the accuracy of the predicted output is, in [30] nonlinear explicit MPC (NEMPC) was used to avoid the computational complexity of iterative online optimization, in [31] the output error state observer was used with MPC to correct the output variable prediction and hence improve the performance of the MPC, also in [32] extended kalman filter was used with NEMPC to estimate unavailable states for T1DP to improve the accuracy of the predicted BGL. To deal with unmeasured state variables, observer was used in conjunction with nonlinear adaptive controller in [33], with backstepping controller in [9], and with predictor feedback controller in [34] to estimate unmeasured state variables. For mentioned control approaches in [9, 31–34] better regulation of BGL was achieved based only on BGL measurements without the need to measure other status variables of remote insulin and plasma insulin level, however the desired performance of the system depends on the accuracy of the estimated state variables which the observer model must be close to the real system. Accordingly, there is a research gap in the literature, is there a control approach that can effectively regulate the BGL of T1DPs to counteract the uncertainties introduced into the system based only on BGL measurements without having to use the system model, nor does it need to measure or estimate other state variables of remote insulin and plasma level. In this regard FLC was applied in [35, 36].

FLC have gained popularity in the last two decades where it doesn’t depend on the mathematical model of the controlled system, it depends on the designer’s previous experiences and his knowledge about the controlled system, this knowledge and experiences about the system are expressed in the form of (IF–THEN) rules, then this rules are incorporated into FLC on how to best control the system, thereby FLC emulate the decision making process of the human that enable us to utilize our previous experience and knowledge about the controlled system [37].

However when the controlled system is exposed to uncertainties, FLC can't deal with it, can't reduce its impact on the controlled system and thus it reduces the desired performance of the system. Due to its internal structure, type-2 fuzzy logic controller (T2FLC) is very appropriate in such situation as it have the capability to model the uncertainties in the system and minimize its impact, thus it improves the performance of the systems that are exposed to uncertainties [38]. There are many sources of uncertainties that affect the system: (1) External disruptions that have an impact on the system's stability because they directly disrupt data transfer from sensors to controllers and from controllers to actuators [39]. (2) The sensors utilized to provide the system with the necessary measurements lack reliability, and as a result, the data based on these measurements is noisy. [40]. (3) Due to the relationships and dependencies among their components, complex nonlinear systems are challenging to represent, which causes modeling uncertainty [41, 42]. (4) We describe variables with words rather than numbers in the antecedent and consequent parts of the rule base of a fuzzy logic system (FLS) based controller, which can be a rich source of uncertainties since words can have various meanings to different people [43]. Moreover, as we mentioned before uncertainties related to the patient like parametric uncertainty (the parameters of the model vary from patient to other as well as the parameters vary within the same patient), uncertain meal disturbance, and physical exercise that can burn glucose, thereby the dose of insulin needs to be continually changed [13].

Over time T2FLC has shown superiority in different applications in dealing with uncertainties and minimizing its impact on the system, therefor in this study an IT2FLC (a special case of T2FLC) is designed to obtaining accurate-safe regulation of BGL for T1DPs during their daily life. The uncertain nonlinear EBMM is used to present glucose-insulin physiological system of T1DP, the parameters of IT2FLC are tuned using GWOCS [44] to infuse the proper amount of exogenous insulin. Then a comparison includes four different optimization methods for IT2FLC such as GWOCS, ALO [45], PSO [46], WOA [47] and FLC method is constructed to show the superiority of IT2FLC using GWOCS over the other methods.

Superiority of IT2FLC in minimizing the effect of uncertainties in the system depends primarily on the best choice of FOU of interval type-2 fuzzy set (T2FS) for each of inputs and output of IT2FLC. FOU enable us to incorporate uncertainties in the membership function of T2FS thereby varying FOU saves a more degree of freedom in designing IT2FLC. FOU can be chosen using an appropriate optimization method, the selection of a good optimization method will improve the performance of IT2FLC. GWOCS is found to be the best optimization method after comparing with other optimization methods as will be shown at rest of this paper. So FOU for each of inputs and output of IT2FLC are tuned using GWOCS where the objective function (OF) of GWOCS is minimized during the optimization process in order to decrease the effect of both parametric uncertainty and uncertain meal disturbance and stabilize BGL within the standard-safe range. Thereby IT2FLC using GWOCS contributes essentially in regulation of BGL for T1DP.

The performance and the effectiveness of the proposed controller are evaluated under six different scenarios of T1DP using Matlab/Simulink platform. Scenario in which nominal parameters of EBMM are used under difficult conditions that T1DP is already in the state of hyperglycemia (BGL > 180 mg/dl) at the start of simulation along with a large meal containing high amount of glucose is provided to T1DP. Other scenario in which a 100 simulation rounds are run to study the behavior of 100 virtual-different T1DPs (parametric uncertainty of EBMM) along with a large meal. Other scenario in which a 100 simulation rounds are run with a 100 virtual-different T1DPs to study the behavior of varying both amount of meal and its time randomly. Also in order to perform a simulation that is very close to the reality, a simulation scenario with 24 h simulation period is performed for nominal parameters of the model in addition to model under the presence of both parametric uncertainty and uncertain meal disturbance for 200 virtual T1DPs with the following configuration: three variable different meals for breakfast, lunch, and dinner are randomly chosen from the range 4 mg/dl/min to 9 mg/dl/min Moreover, other critical scenario with the configuration of three different meals 5 mg/dl/min for breakfast, 8 mg/dl/min for lunch and dinner are provided at 8 am, 12 am, and 8 am respectively along with random initial condition of all the state variables of EBMM. Also, a graphical tool called control variability grid analysis (CVGA) is used to assessment the quality of the proposed controller [48].

Simulation results show that the proposed controller can avoid both severe hypoglycemia and hyperglycemia for nominal parameters of the model, in addition to model under the presence of both parametric uncertainty and uncertain meal disturbance and random initial condition of the state variables of EBMM. Control signal of the proposed controller is smooth, not aggressive and always non-negative, it doesn't need any excessive method or algorithm to inject glucagon or glucose to avoid hypoglycemia [49]. Consequently the proposed controller is less complex, low cost and easy to implement. Also stability analysis for the system is performed using Lyapunov function to determine the stability region of nonlinear system and simulation results of scenario1 and scenario 2 of Sect. 3 prove that the system operates within this stability region that its constraints are deduced in Sect. 2.

The main contributions of this work are as follows:

- Here, an efficient method for regulation of BGL for T1DP is presented using IT2FLC with the conjunction of different optimization methods such as GWOCS, ALO, PSO, and WOA, then a comparison includes these optimization methods and FLC method is constructed to select the best controller that achieves the best results to infuse the proper amount of exogenous insulin. Importantly, the combination of IT2FLC and multiple optimization methods is a novel approach for regulation BGL that has not been explored in previous works. These contributions demonstrate the potential of the proposed method to significantly improve glucose control in type 1 diabetic patients.
- The tuning of parameters was done for range of FOU of each of inputs and output of IT2FLC but the scaling factors of IT2FLC are held constant and equal to 1. This will increase the robustness of the controller against of the uncertainties.
- Although the nonlinearities are considered in the model, the controller is easy to implement and the performance of the controller is investigated under different scenarios and conditions of T1DPs to prove the effectiveness of the proposed technique. Also CVGA plot is used to assessment the quality of the proposed controller for these scenarios.

The rest of this paper is categorized as follow: Section 2 presents and details the following points, the mathematical model of EBMM and the meaning of its variables and parameters, the concept of type-2 fuzzy logic system (T2FLS) and T2FS with a detailed explanation of how to proceed into each stage of interval type-2 fuzzy logic system, design of IT2FLC for regulation of BGL for T1DP with a detailed explanation of GWOCS, and Stability analysis for the system using Lyapunov function to determine the stability region of nonlinear system. In Sect. 3 results and discussion are presented. Finally conclusion is presented in Sect. 4.

## Materials and methods

### EBMM of T1DP

Because it is less complex than the others and keeps the minimal state variables necessary to describe the relationship between glucose and insulin for diabetic patient, an intravenous glucose tolerance test (IVGTT) of the Bergman minimal (BM) model [15] is regarded as the most popular in research on artificial pancreas. The three differential equations with its states will model the complete behavior of glucose and insulin kinetics for patients. The first one indicates BGL, where the other one reveals the remote insulin, and the last equation indicates plasma insulin level.

To model the behavior of meal disturbance in BM model, a new equation with a new state variable has been added in the extended Bergman minimal model (EBMM) to constitute the dynamics of meal [33].

Thereby the mathematical equations of the EBMM are as follow and also the meaning of variables and parameters of EBMM are explained in Table 1:

1 $$\dot{x}_{1} = - p_{1} \left( {x_{1} - G_{b} } \right) - x_{1} x_{2} + x_{4}$$

2 $$\dot{x}_{2} = - p_{2} x_{2} + p_{3} \left( {x_{3} - I_{b} } \right)$$

3 $$\dot{x}_{3} = - p_{4} \left( {x_{3} - I_{b} } \right) + u\left( t \right)$$

4 $$\dot{x}_{4} = - p_{5} x_{4}$$

**Table 1:** Meaning of variables and parameters of EBMM

Variables and parameters	Meaning of variables and parameters	Dimension
x_1_	BGL	mg/dl
x_2_	Delayed effect of insulin on BGL	min^−1^
x_3_	plasma insulin level	mU/l
x_4_	the dynamics of meal disturbance, its initial state represents amount of glucose in the meal	mg/dl/min
u(t)	rate of exogenous insulin infusion which replaces the natural insulin in the normal body	mU/l/min
G_b_	basal value of BGL at which the BGL stabilizes after glucose is absorbed	mg/dl
I_b_	basal value of plasma insulin level at which insulin level stabilizes after insulin is released to lower BGL in blood stream	mU/l
p_1_	Insulin-independent glucose utilization, its value approaches to zero for T1DPs	min^−1^
$\frac{{{\text{p}}_{3} }}{{{\text{p}}_{2} }}$	insulin sensitivity	$\frac{{\text{l}}}{{\text{mU }}}$
p_4_	insulin degradation rate	min^−1^
p_5_	rate of appearance of meal disturbance in plasma glucose	min^−1^

### Type-2 fuzzy logic system

T2FLC is a T2FLS based controller, the superiority of T2FLS over FLS comes through its ability to model uncertainties about any variable in the system and minimize its impact, that's why the benefits of T2FLS appear strongly in applications that are exposed to uncertainties. Modeling uncertainties needs a method to measure dispersion about the variable (how the points that constitute the uncertainties about the variable are distributed and spanned) [50]. So a set of numbers is needed rather than a single specific number in FLS to capture more information about uncertainties about any variable in the system. T2FLS saves this method to model uncertainties and can effectively minimize its impact while the ordinary FLS has limited capabilities in this regard [50].

**Fig. 2:**
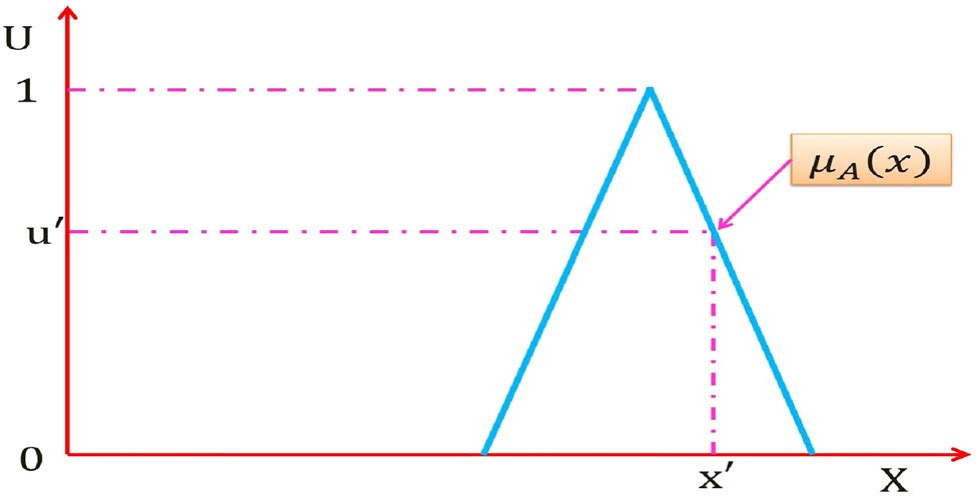
FS of an ordinary FLS

Figure 2 shows fuzzy set (FS) denotes "A" of an ordinary FLS, the membership grade of any given point $x^{\prime } \in X$ on the universe of discourse *X* equal to *u*′ which is a single specific number.

The FS "A" can be expressed mathematically as:

5 $$A = \left\{ {\left( {x,\mu _{A} \left( x \right)} \right)\quad \forall x \in X} \right\}\quad {\text{Or}}\quad A = \int\limits_{{x \in X}} {\frac{{\mu _{A} \left( x \right)}}{x}}$$

$\smallint$ Denotes union over all admissible x, *μ*_*A*_(*x*) is the membership grade of *x* ∈ *X* in *A* and it takes just one value from the constraint $0 \le \mu_{A} \left( x \right) \le 1$.

**Fig. 3:**
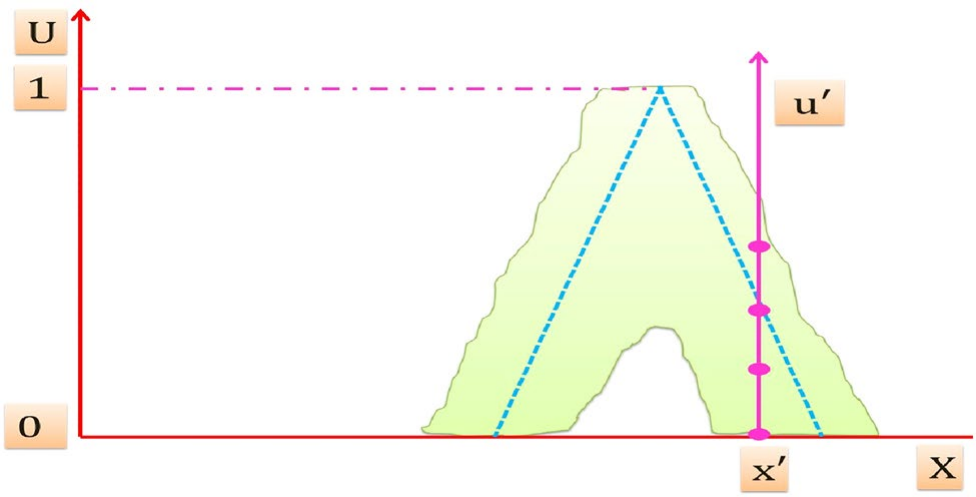
Blurred MF of FS of an ordinary FLS

**Fig. 4:**
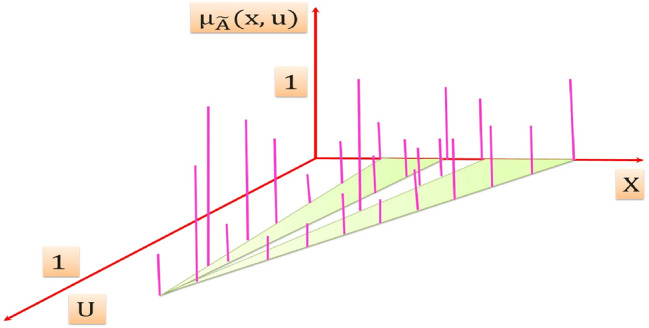
Three dimension T2MF

A T2FS denotes "$\tilde{A}$ " of T2FLS is depicted in Fig. 3. It is resulted from blurring the membership function (MF) of FS of Fig. 2 [51], the membership grade of *x*′ on T2FS is a set of numbers takes their values from the intersection of the vertical line at *x*′ in the U axis. It can be assigned at each point resulted from the intersection of the vertical line at *x*′ an amplitudes distribution that can construct a three dimension type-2 membership function (T2MF) as shown in Fig. 4, its axis are $x, u, \mu_{{\tilde{A}}} \left( {x,u} \right)$ which characterizes a T2FS. Those amplitudes distribution in $\mu_{{\tilde{A}}} \left( {x,u} \right)$ axis is not necessary that all of them be equal.

"$\tilde{A}$" can be expressed mathematically as [51]:

6 $$\tilde{A} = \left\{ {\left( {\left( {x,u} \right),\mu_{{\tilde{A}}} \left( {x,u} \right)} \right) \quad \forall x \in X,\quad \forall u \in J_{x} \left[ {0, 1} \right]} \right\}\quad {\text{Or}}\quad \tilde{A} = \mathop \smallint \limits_{x \in X} \mathop \smallint \limits_{u \in U} \frac{{\mu_{{\tilde{A}}} \left( {x,u} \right)}}{{\left( {x,u} \right)}} u \in J_{x} \left[ {0,1} \right]$$

${\iint }$ Denotes union over all admissible *x* and *u*, and $\mu_{{\tilde{A}}} \left( {x,u} \right)$ is a T2MF that characterizes a T2FS, it takes their values from the constraint $0 \le \mu_{{\tilde{A}}} \left( {x,u} \right) \le 1$.

*J*_*x*_ Is the primary membership of *x* in $\tilde{A}$, also called the domain of a secondary membership of *x* in $\tilde{A}$.

The membership grades of the primary membership of *x* in $\tilde{A}$ are called the secondary membership of *x* in $\tilde{A}$ and also called the vertical slices in $\mu_{{\tilde{A}}} \left( {x,u} \right)$ axis.

**Fig. 5:**
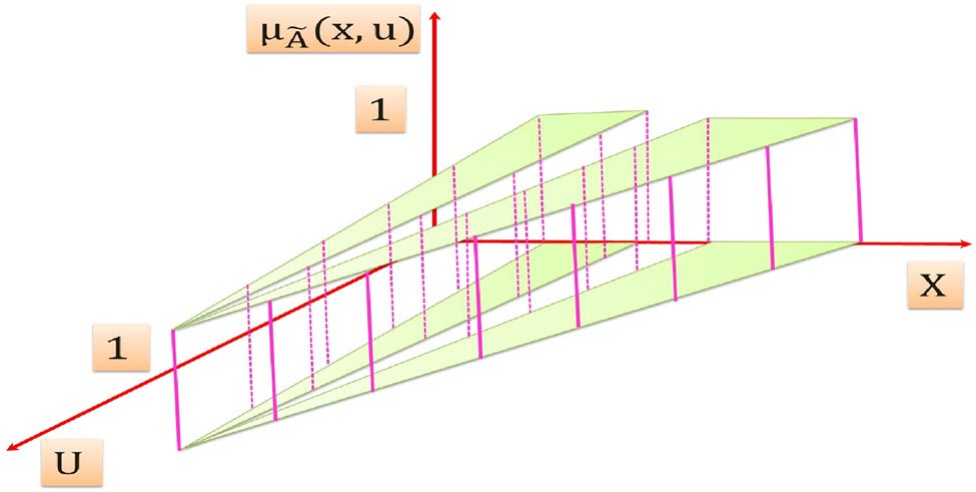
The IT2MF

When all of the vertical slices in $\mu_{{\tilde{A}}} \left( {x,u} \right)$ axis equal 1, then the resulting T2MF is an interval type-2 membership function (IT2MF) as shown in Fig. 5 that characterizes an interval type-2fuzzy set (IT2FS) [52].

$\tilde{A}$ Can be expressed mathematically as [52]:

7 $$\tilde{A} = \int\limits_{{x \in X}} {\int\limits_{{u \in U}} {\frac{1}{{\left( {x,u} \right)}}J_{x} \left[ {0,1} \right]} }$$

The shaded region on the *x* − *u* plane in Fig. 5 is the FOU, the FOU is a complete description of an IT2FS, this is because the secondary grades of IT2FS equal to 1, and thereby it conveys no new information [52]

The FOU in the primary memberships of $\tilde{A}$ is the union of all primary memberships that is:

8 $$FOU\left( {\tilde{A}} \right) = \bigcup\nolimits_{x \in X} {J_{x} }$$

**Fig. 6:**
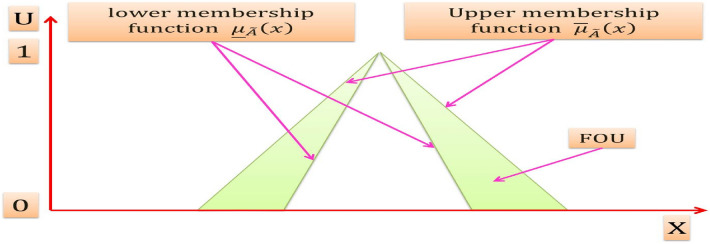
FOU for an IT2FS

The FOU for an IT2FS is shown in Fig. 6, it is bounded from the above and below by a two ordinary MFs that are called lower MF and upper MF, and are denoted $\underline{{\mu_{{\tilde{A}}} }} \left( x \right),\overline{{\mu_{{\tilde{A}}} }} \left( x \right),\forall x \in X$ respectively.

#### Interval type-2 fuzzy logic system

Over the years a lot of efforts have been made to facilitate the complex mathematical operations accompanying T2FLS so that researchers and engineers can utilize it in solving problems of many different applications in various areas [53]. The mathematical operations accompanying T2FSs are very complex and time consuming while it is simple to IT2FSs (IT2FSs is considered a special case of general T2FSs)[52], since it needs to can use IT2FSs that we are just familiar with the mathematical operations of ordinary FSs such as union, intersection, and complement. That is why IT2FLS that is characterized by its IT2FSs have gained popularity among many practitioners and engineers. Consequently IT2FLS have been contributed in different applications: in [54] it controls restricted crossing U-turn traffic, in [55] it simulates the driver's operating habits of intelligent vehicles control, in [56] its application in handling map matching uncertainties for airport ground movements, and In [57] regulate the mean arterial blood pressure for hypertensive patients with different health status conditions.

**Fig. 7:**
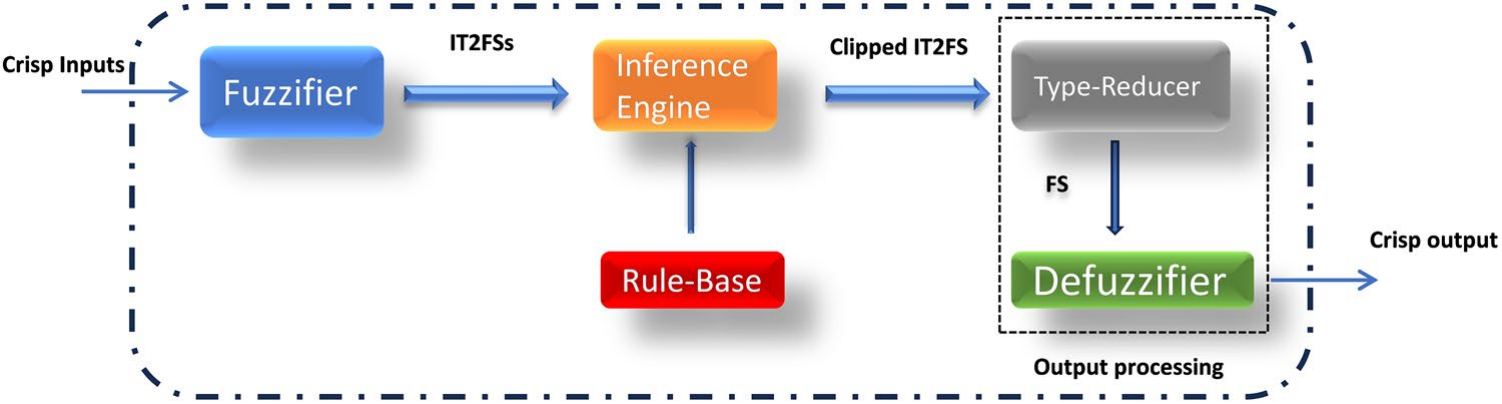
The structure of IT2FLS

The structure of IT2FLS is shown in Fig. 7 [38]. It is almost the same as for ordinary FLS, its rule-base is composed of (IF–THEN) rules as for FLS but now some or all of its antecedent and consequent parts are of IT2FS. It differs from FLS in that the output of the fuzzifier block is IT2FSs, consequently the fuzzy output sets of inference engine block is IT2FSs. Since most applications like in a control system require a single crisp number from the output of the controller rather than a set of numbers in order to can take a control action, the defuzzifier block in FLS is replaced by output processing block which internally consists of two blocks the first which converts IT2FS to FS that is called type-reducer and the second is just a defuzzifier as in FLS to convert FS to a single crisp number.

There are many algorithms one can use to perform type-reduction, Karnik–Mendel (KM) algorithm [58] is the popular one it is simple and converges to the exact solution but it is iterative and computationally intensive. So in this paper we will utilize enhanced iterative algorithm with stop condition (EIASC) to perform type-reduction as EIASC can save more 50% computational cost over KM algorithm particularly when number of rules *N* ≤ 100 [59].

Now in the following subsections we will explain in details how to proceed in each stage of IT2FLS that will be incorporated into IT2FLC to obtain accurate-safe regulation of BGL for T1DPs.

#### Rule-base of ITFLS

Zadeh rule-base is considered in this paper, the structure of rule-base for IT2FLS remains the same as for FLS. The only difference is some or all of its antecedent and consequent parts are of IT2FS, the structure of the general Zadeh rule for IT2FLS which has *p* inputs $x_{1} \in X_{1} , \ldots .,x_{p} \in X_{p}$, and one output *y* ∈ *Y* is as follow [52]:

$$R^{l} {:}IF\,x_{1} \,is\,\tilde{F}_{1}^{l} \quad and \cdots and\quad x_{p } \,is\,\tilde{F}_{p}^{l} ,\, THEN\,y\,is\,\tilde{G}^{l} ,\quad l = 1, \ldots ., N.$$

where *N* is number of rules, *l* is the rank of rule, $\tilde{F}_{1}^{l} \ldots \ldots ..\tilde{F}_{p}^{l}$ are IT2FSs, and $\tilde{G}^{l}$ is IT2FS which is an interval set is expressed in the form of endpoints centroid of a consequent of IT2FS [34], where $\tilde{G}^{l} = \left[ {\underline {G}^{l} ,\underline {G}^{l} } \right]$.

**Fig. 8:**
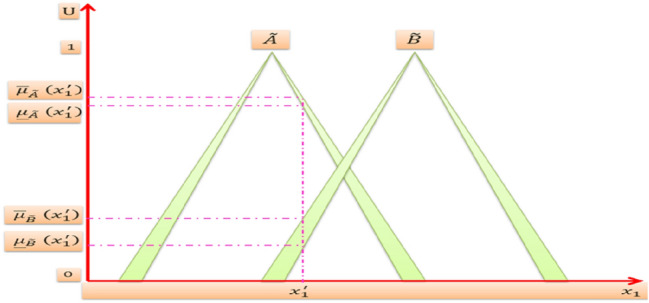
IT2MFs of input ***x***_1_

**Fig. 9:**
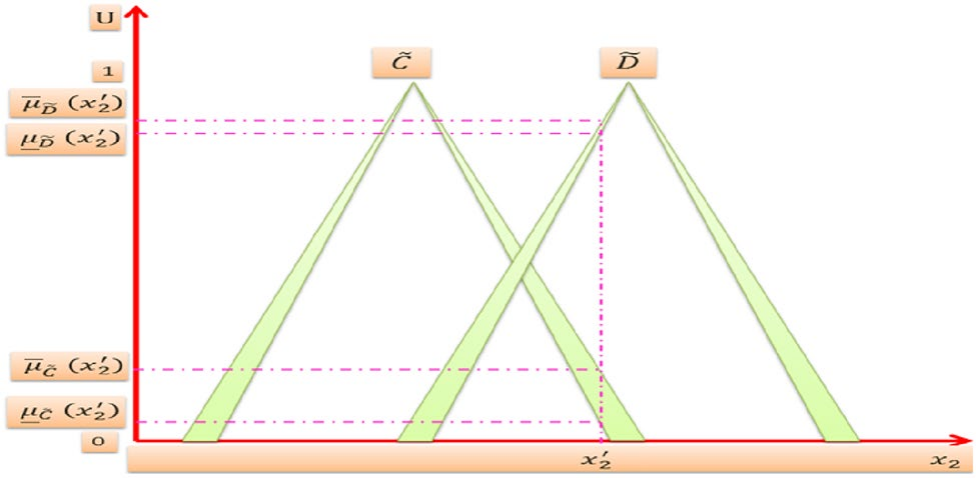
IT2MFs of input ***x***_2_

**Fig. 10:**
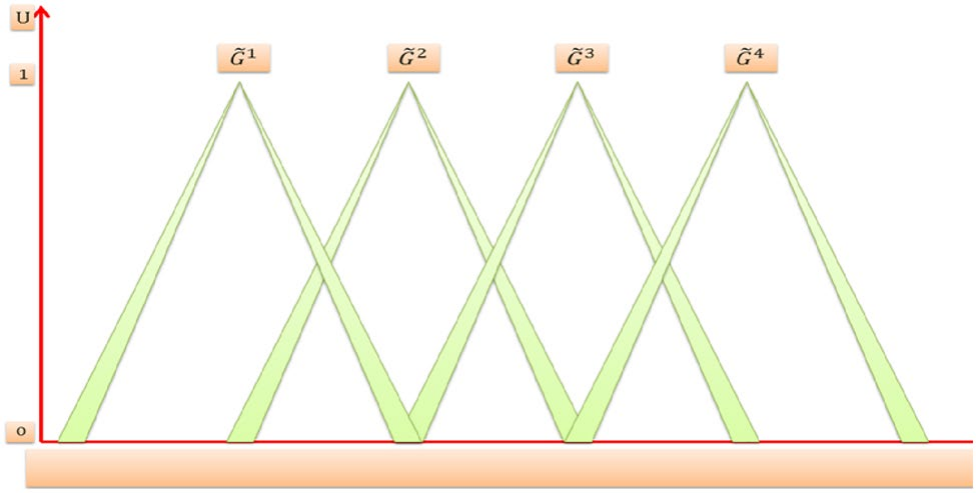
IT2MFs of output ***y***

Suppose we have an IT2FLS with four rules *N* = 4, two inputs *x*_1_, *x*_2_ and one output *y*. The domain of input *x*_1_ consists of two triangular IT2MFs $\tilde{A}$ and $\tilde{B}$ as shown in Fig. 8. The domain of input *x*_2_ consists of two triangular IT2MFs $\tilde{C}$ and $\tilde{D}$ as shown in Fig. 9. And the domain of output *y* consists of four triangular IT2MFs $\tilde{G}^{1} , \tilde{G}^{2} ,\tilde{G}^{3}$, and $\tilde{G}^{4}$ as shown in Fig. 10, then the four rules are:

$$\begin{aligned} & R^{1} {:}IF\,x_{1} \,is\,\tilde{A}\quad and\quad x_{2} \,is\,\tilde{C},\,THEN\,y\,is\,\tilde{G}^{1} \\ & R^{2} {:}IF\,x_{1} \,is\,\tilde{A}\quad and\quad x_{2} \,is\,\tilde{D},\,THEN\,y\,is\,\tilde{G}^{2} \\ & R^{3} {:}IF\,x_{1} \,is\,\tilde{B}\quad and\quad x_{2} \,is\,\tilde{C},\,THEN\,y\,is\,\tilde{G}^{3} \\ & R^{4} {:}IF\,x_{1} \,is\,\tilde{B}\quad and\quad x_{2} \,is\,\tilde{D},\, THEN\,y\,is\,\tilde{G}^{4} \\ \end{aligned}$$

#### Fuzzifier of IT2FLS

It performs fuzzification process which converts crisp inputs into IT2FSs. It is the same as for the fuzzification of ordinary FSs, but now we have for each point in the universe of discourse two interval membership values rather than one membership value in the FSs, both of them are calculated from the intersection of the vertical line with the lower MF and upper MF respectively. For example when $x_{1} = x_{1}^{\prime }$ and $x_{2} = x_{2}^{\prime }$, then the vertical line at $x_{1}^{\prime }$ intersects $\tilde{A}$ within the two interval values $\left[ {\underline {\mu }_{{\tilde{A}}} \left( {x_{1}^{\prime } } \right),\overline{\mu }_{{\tilde{A}}} \left( {x_{1}^{\prime } } \right)} \right]$ also intersects $\tilde{B}$ within the two interval values $\left[ {\underline {\mu }_{{\tilde{B}}} \left( {x_{1}^{\prime } } \right),\overline{\mu }_{{\tilde{B}}} \left( {x_{1}^{\prime } } \right)} \right]$. The vertical line at $x_{2}^{\prime }$ intersects $\tilde{C}$ within the two interval values $\left[ {\underline {\mu }_{{\tilde{C}}} \left( {x_{2}^{\prime } } \right),\overline{\mu }_{{\tilde{C}}} \left( {x_{2}^{\prime } } \right)} \right]$ also intersects $\tilde{D}$ within the two interval values $\left[ {\underline {\mu }_{{\tilde{D}}} \left( {x_{2}^{\prime } } \right),\overline{\mu }_{{\tilde{D}}} \left( {x_{2}^{\prime } } \right)} \right]$. Thereby the interval values that deduced from the fuzzification process of the inputs are:

$$\begin{aligned} \mu_{{\tilde{A}}} \left( {x_{1}^{\prime } } \right) & = \left[ {\underline {\mu }_{{\tilde{A}}} \left( {x_{1}^{\prime } } \right),\overline{\mu }_{{\tilde{A}}} \left( {x_{1}^{\prime } } \right)} \right] \\ \mu_{{\tilde{B}}} \left( {x_{1}^{\prime } } \right) & = \left[ {\underline {\mu }_{{\tilde{B}}} \left( {x_{1}^{\prime } } \right),\overline{\mu }_{{\tilde{B}}} \left( {x_{1}^{\prime } } \right)} \right] \\ \mu_{{\tilde{C}}} \left( {x_{2}^{\prime } } \right) & = \left[ {\underline {\mu }_{{\tilde{C}}} \left( {x_{2}^{\prime } } \right),\overline{\mu }_{{\tilde{C}}} \left( {x_{2}^{\prime } } \right)} \right] \\ \mu_{{\tilde{D}}} \left( {x_{2}^{\prime } } \right) & = \left[ {\underline {\mu }_{{\tilde{D}}} \left( {x_{2}^{\prime } } \right),\overline{\mu }_{{\tilde{D}}} \left( {x_{2}^{\prime } } \right)} \right] \\ \end{aligned}$$

#### Inference engine of IT2FLS

It deduces the clipped IT2FS consequent for the output variable of each rule in the rule-base for a given crisp value of the input variables (those values that we get from the fuzzifier of ITFLS stage) and this process also called rule firing. Consequently we have for each rule of IT2FLS two interval firing level, lower firing level $\underline {f}^{l}$ and upper firing level $\overline{ f}^{l}$ [38, 50]. Then it combines all the clipped IT2FSs consequents of the fired rules into one overall IT2FS that constitutes the output of the inference engine which is the input to the output processing block. Interval type-2 mamdani fuzzy system and minimum t-norm operator are considered, so the two firing interval for the four rules are:

$$\begin{aligned} \left[ {\underset{\raise0.3em\hbox{$\smash{\scriptscriptstyle-}$}}{f} ^{1} ,\bar{f}^{1} } \right] &= \left[ {min\left[ {\underset{\raise0.3em\hbox{$\smash{\scriptscriptstyle-}$}}{\mu } _{{\tilde{A}}} \left( {x_{1} ^\prime } \right),\underset{\raise0.3em\hbox{$\smash{\scriptscriptstyle-}$}}{\mu } _{{\tilde{C}}} \left( {x_{2} ^\prime } \right)} \right],min\left[ {\bar{\mu }_{{\tilde{A}}} \left( {x_{1} ^\prime } \right),\bar{\mu }_{{\tilde{C}}} \left( {x_{2} ^\prime } \right)} \right]} \right]\\ \left[ {\underset{\raise0.3em\hbox{$\smash{\scriptscriptstyle-}$}}{f} ^{2} ,\bar{f}^{2} } \right]& = \left[ {min\left[ {\underset{\raise0.3em\hbox{$\smash{\scriptscriptstyle-}$}}{\mu } _{{\tilde{A}}} \left( {x_{1} ^\prime } \right),\underset{\raise0.3em\hbox{$\smash{\scriptscriptstyle-}$}}{\mu } _{{\tilde{D}}} \left( {x_{2} ^\prime } \right)} \right],min[\bar{\mu }_{{\tilde{A}}} \left( {x_{1} ^\prime } \right),\bar{\mu }_{{\tilde{D}}} \left( {x_{2} ^\prime } \right)} \right]]\\\left[ {\underset{\raise0.3em\hbox{$\smash{\scriptscriptstyle-}$}}{f} ^{3} ,\bar{f}^{3} } \right] &= \left[ {min\left[ {\underset{\raise0.3em\hbox{$\smash{\scriptscriptstyle-}$}}{\mu } _{{\tilde{B}}} \left( {x_{1} ^\prime } \right),\underset{\raise0.3em\hbox{$\smash{\scriptscriptstyle-}$}}{\mu } _{{\tilde{C}}} \left( {x_{2} ^\prime } \right)} \right],min\left[ {\bar{\mu }_{{\tilde{B}}} \left( {x_{1} ^\prime } \right),\bar{\mu }_{{\tilde{C}}} \left( {x_{2} ^\prime } \right)} \right]} \right]\\ \left[ {\underset{\raise0.3em\hbox{$\smash{\scriptscriptstyle-}$}}{f} ^{4} ,\bar{f}^{4} } \right] &= \left[ {min\left[ {\underset{\raise0.3em\hbox{$\smash{\scriptscriptstyle-}$}}{\mu } _{{\tilde{B}}} \left( {x_{1} ^\prime } \right),\underset{\raise0.3em\hbox{$\smash{\scriptscriptstyle-}$}}{\mu } _{{\tilde{D}}} \left( {x_{2} ^\prime } \right)} \right],min\left[ {\bar{\mu }_{{\tilde{B}}} \left( {x_{1} ^\prime } \right),\bar{\mu }_{{\tilde{D}}} \left( {x_{2} ^\prime } \right)} \right]} \right]\end{aligned}$$

**Fig. 11:**
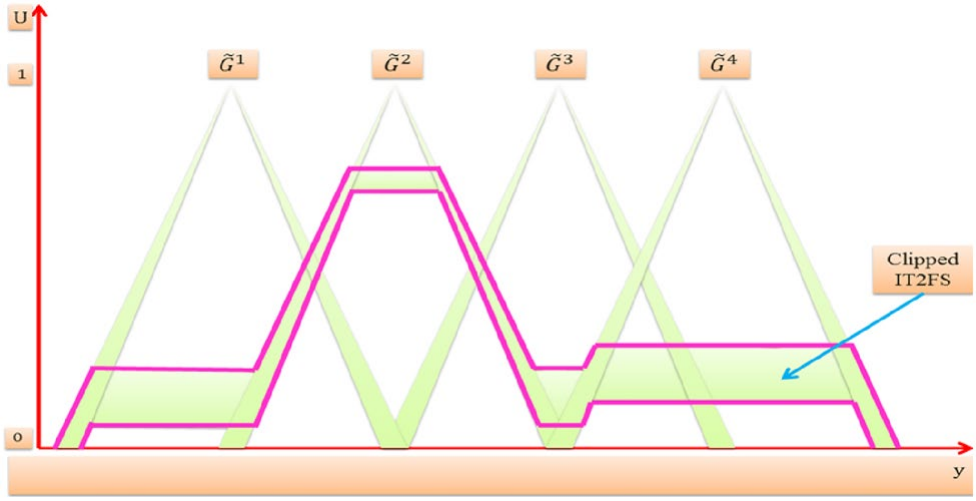
One overall clipped IT2FS

Now we have got the firing interval of each rule, then $\underline {f}^{l}$ for each rule is t-normed with lower membership function of $\tilde{G}^{l}$ and $\overline{ f}^{l}$ for each rule is t-normed with upper membership function of $\tilde{G}^{l}$ to get a clipped IT2FS consequent from each rule. After that the clipped IT2FS consequent of each rule are combined together (using union operator) into one overall clipped IT2FS as shown in Fig. 11.

#### Type-reduction and defuzzification of IT2FSs

It was supposed just as for FLS after we got the resulted clipped IT2FS that we can as usual run one of the defuzzification methods to get the crisp output. But for the IT2FLS the union of the clipped IT2FSs of each rule becomes a big issue with regarding to the computational complexity as it requires additional computational time and memory storage particularly with real time applications such as a control system [38, 50].

So instead of that type-reducer + defuzzification method is used to combine the firing interval of each rule with the corresponding rule consequent and get the crisp output. There are many methods to do that: Centroid, Height, and Center-of-sets. In this paper we will use center-of-sets (COS) method for an interval type-2 mamdani fuzzy system in which the firing interval of each rule is combined with the corresponding endpoints centroids of its consequent [38, 58].

By using this method we get the COS type-reduced-set denoted *Y*_*cos*_(*x*′) for an interval type-2 mamdani fuzzy system Where:

9 $$Y_{cos} \left( {x^{\prime } } \right) = 1/\left[ {y_{l} ,y_{r} } \right],$$

10 $$y_{l} = \frac{{\mathop \sum \nolimits_{l = 1}^{L} \overline{f}^{l} \underline {G}^{l} + \mathop \sum \nolimits_{l = L + 1}^{N} \underline {f}^{l} \underline {G}^{l} }}{{\mathop \sum \nolimits_{l = 1}^{L} \overline{f}^{l} + \mathop \sum \nolimits_{l = L + 1}^{N} \underline {f}^{l} }},$$

11 $$y_{r} = \frac{{\mathop \sum \nolimits_{l = 1}^{R} \underline {f}^{l} \overline{G}^{l} + \mathop \sum \nolimits_{l = R + 1}^{N} \overline{f}^{l} \overline{G}^{l} }}{{\mathop \sum \nolimits_{l = 1}^{R} \underline {f}^{l} + \mathop \sum \nolimits_{l = R + 1}^{N} \overline{f}^{l} }},$$

N is number of rules, L and R are the switch points that.

Satisfy:

$$\begin{aligned} \underline {G}^{L} & \le y_{l} \le \underline {G}^{L + 1} \\ \overline{G}^{R} & \le y_{r} \le \overline{G}^{R + 1} \\ \end{aligned}$$

*y*_*l*_ and *y*_*r*_ can be computed using EIASC algorithm [59], after that we can easily get the crisp output of interval type-2 mamdani fuzzy system from the following formula

12 $$y = \frac{{y_{l} + y_{r} }}{2}.$$

But before we go through EIASC algorithm we have to first calculate for each rule the endpoints centroids of its consequent by using the KM algorithm which is calculated only once [58] as follow:

$$\begin{aligned} \tilde{G}^{1} & = \left[ {\underline {G}^{1} , \overline{G}^{1} } \right] \\ \tilde{G}^{2} & = \left[ {\underline {G}^{2} , \overline{G}^{2} } \right] \\ \tilde{G}^{3} & = \left[ {\underline {G}^{3} , \overline{G}^{3} } \right] \\ \tilde{G}^{4} & = \left[ {\underline {G}^{4} , \overline{G}^{4} } \right] \\ \end{aligned}$$

where $\underline {G}^{l} ,\overline{G}^{l}$ are the two endpoints centroids of the consequent $\tilde{G}^{l}$ and $l = 1, \ldots \ldots ,N$.

EIASC algorithm for computing *y*_*l*_ [59]:

- Sort $\underline {G}^{l} {\kern 1pt} \left( {l = 1, \ldots \ldots ,N} \right)$ in increasing order and call the sorted $\underline {G}^{l}$ by the same name, but now $\underline {G}^{1} \le \underline {G}^{2} \le \cdots \le \underline {G}^{N}$. Match the weights *F*^*l*^ (*x*′) with their respective $\underline {G}^{l}$ and renumber them so that their index corresponds to the renumbered $\underline {G}^{l}$.
- Initialize
  $$\begin{aligned} a & = \mathop \sum \limits_{l = 1}^{N} \underline {G}^{l} \underline {f}^{l} , \\ b & = \mathop \sum \limits_{l = 1}^{N} \underline {f}^{l} , \\ y_{l} & = \underline {G}^{N} ,\quad L = 0. \\ \end{aligned}$$
- Compute
  $$\begin{aligned} L & = L + 1, \\ a & = a + \underline {G}^{L} \left( {\overline{f}^{L} - \underline {f}^{L} } \right), \\ b & = b + \overline{f}^{L} - \underline {f}^{L} , \\ y_{l} & = \frac{a}{b}. \\ \end{aligned}$$
- If $y_{l} \le \underline {G}^{L + 1}$, stop; otherwise go to step (c).
  EIASC algorithm for computing *y*_*r*_ [59]:
  - Sort $\overline{G}^{l} \left( {l = 1, \ldots \ldots ,N} \right)$ in increasing order and call the sorted $\overline {G}^{l}$ by the same name, but now $\overline{G}^{1} \le \overline{G}^{2} \le \cdots \le \overline{G}^{N}$. Match the weights *F*^*l*^(*x*′) with their respective $\overline{G}^{l}$ and renumber them so that their index corresponds to the renumbered $\overline{G}^{l}$.
  - Initialize
    $$\begin{aligned} a & = \mathop \sum \limits_{l = 1}^{N} \overline{G}^{l} \underline {f}^{l} , \\ b & = \mathop \sum \limits_{l = 1}^{N} \underline {f}^{l} , \\ y_{r} & = \overline{G}^{1} ,\quad R = N. \\ \end{aligned}$$
  - Compute
    $$\begin{aligned} a & = a + \overline{G}^{R} \left( {\overline{f}^{R} - \underline {f}^{R} } \right), \\ b & = b + \overline{f}^{R} - \underline {f}^{R} , \\ y_{r} & = \frac{a}{b}, \\ R & = R - 1. \\ \end{aligned}$$
  - If $y_{r} \ge \overline{G}^{R}$, stop; otherwise go to step (c).
  The defuzzified crisp output of interval type-2 Mamdani fuzzy system is:
  $$y = \frac{{y_{l} + y_{r} }}{2}$$

### Design of IT2FLC for regulation of BGL for T1DP

**Fig. 12:**
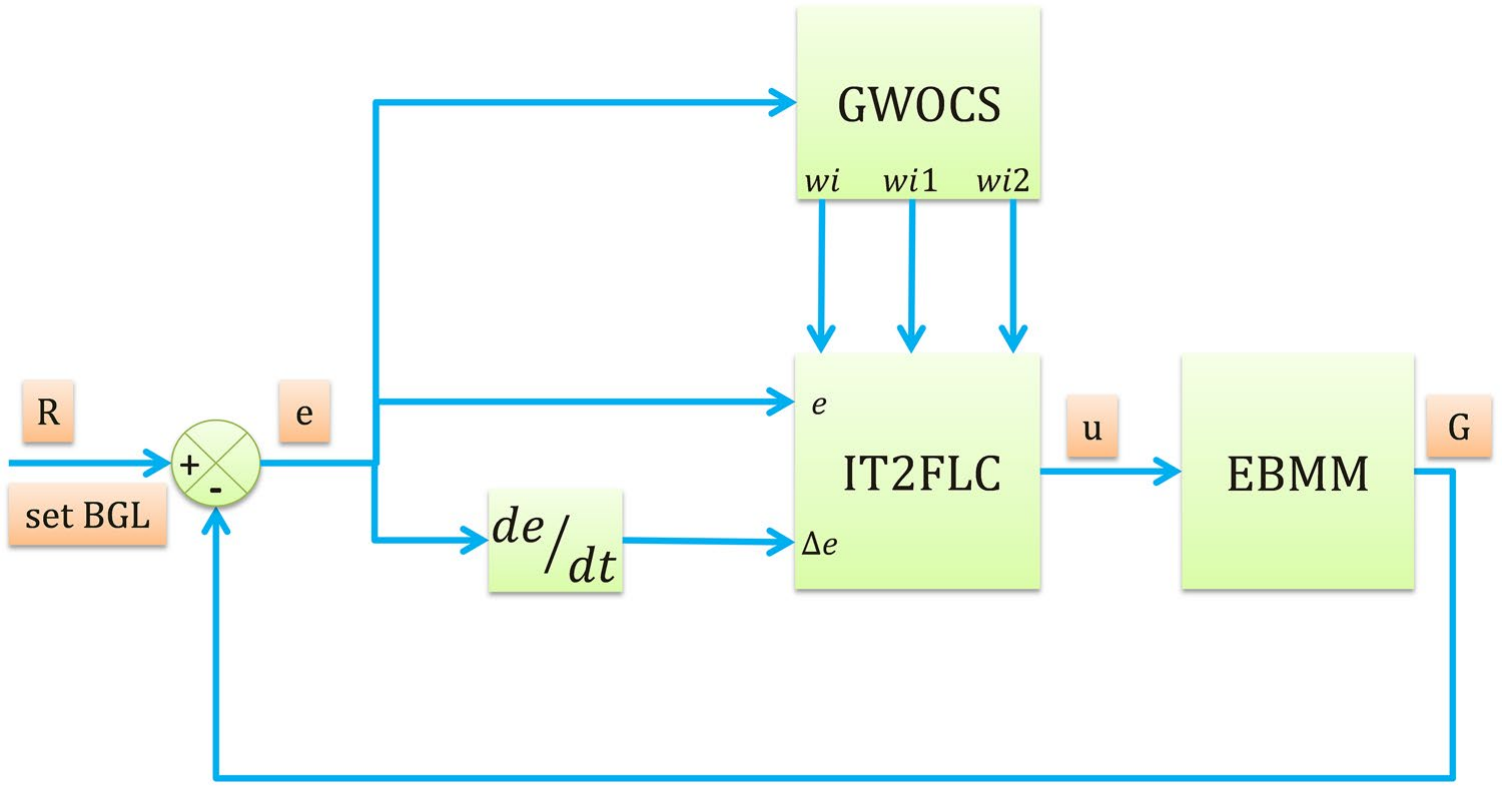
Block diagram of IT2FLC for T1DP

Figure 12 shows the block diagram of IT2FLC for regulation of BGL for T1DP, the inputs to controller are the error signal *e* and the change of error signal Δ*e*, *u* is the output of controller, *R* refers to the desired BGL, and *G* refers to the measured BGL for T1DP.

13 $$u = f\left( {e,\Delta e} \right)$$

14 $$e = R - G$$

**Fig. 13:**
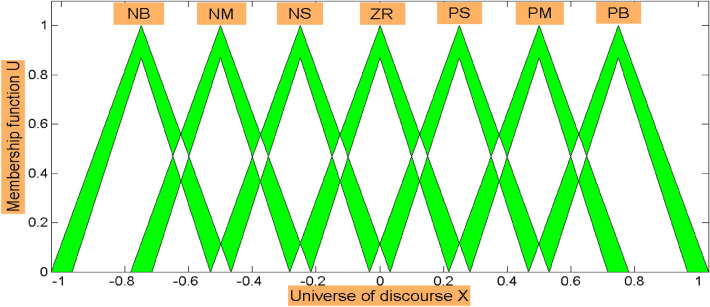
IT2MFs for the error, change of the error, and the output signal

Seven IT2MFs of type Triangular are used for the error signal, change of the error signal and for the output signal as shown in Fig. 13 which are: Negative Big (NB), Negative Medium (NM), Negative Small (NS), Zero (ZR), Positive Small (PS), Positive Medium (PM), and Positive Big (PB).

Table 2 represents the knowledge base defining the rules for the desired relationship between the inputs and output.

**Table 2:** Rule base of error and change of error

Error	Change of error						
	NB	NM	NS	ZE	PS	PM	PB
NB	PB	PB	PB	PB	PM	PS	ZR
NM	PB	PB	PB	PM	PS	ZR	NS
NS	PB	PB	PM	PS	ZR	NS	NM
ZE	PB	PM	PS	ZR	NS	NM	NB
PS	PM	PS	ZR	NS	NM	NB	NB
PM	PS	ZR	NS	NM	NB	NB	NB
PB	ZR	NS	NM	NB	NB	NB	NB

Each of the signals *e*, Δ*e*, *u* has its own FOU where *wi*, *wi*1, *wi*2 in Fig. 12 refer to FOU of IT2MF for *e*, Δ*e*, *u* respectively. We can tune FOU of each signal using GWOCS [44] to obtain *wi*, *wi*1, *wi*2.

During the optimization process the OF is minimized to meet the standard-safe response of BGL, The OF of GWOCS is chosen to be equal to mean absolute of error (MAE) signal.

15 $$OF = \frac{{\mathop \sum \nolimits_{n = 1}^{N} \left| {e\left( t \right)} \right|}}{N}$$

where N is sampling time.

#### Hyprid Grey wolf and Cuckoo search optimizer

In nature, the herd of grey wolves is divided into four main types, alpha, beta, delta, and omega [44]. The herd's alpha is regarded as the one in charge of making decisions. The beta is most likely the best choice to take over as alpha in the event that one of the other wolves passes away or gets too old. Beta supports alpha making its decisions, improves the order of alphas throughout the herd and provide alpha with feedback. Delta is a subordinate to alpha and beta, it supports alpha and beta during the hunting process, it governs and gives orders to omega wolves. Omega is the grey wolf with the lowest ranking in the herd. The omega serves as the scapegoat. They are the final wolves that are permitted to feed. The omega may not seem like a significant member of the herd, however it helps keep the dominance structure of the herd.

The hunting process of grey wolf in nature is divided into three main phases:

- Exploring the position of prey, tracking it, and getting close to the prey.
- Encircling the prey, and harassing it until it stops moving.
- Attacking the prey.

In this study *wi*, *wi*1, *wi*2, the parameters of IT2FLC are tuned using GWOCS algorithm [44], it simulates the hunting process of grey wolf in nature for searching the prey with the conjunction of Cuckoo Search algorithm which is considered one of the meta-heuristic algorithms that draws its inspiration from nature. The population-based stochastic optimization technique known as CS is very effective at searching. The CS has memory automation that can assist in helping to record the neighborhood minimum and contribute in choosing the best. As a result, CS algorithm can more effectively than other techniques search through the designated search space for the most ideal value. Due to its varied lifestyles and encroachment on reproductivity, the cuckoo bird is a type of bird that CS algorithm draws inspiration.

**Fig. 14:**
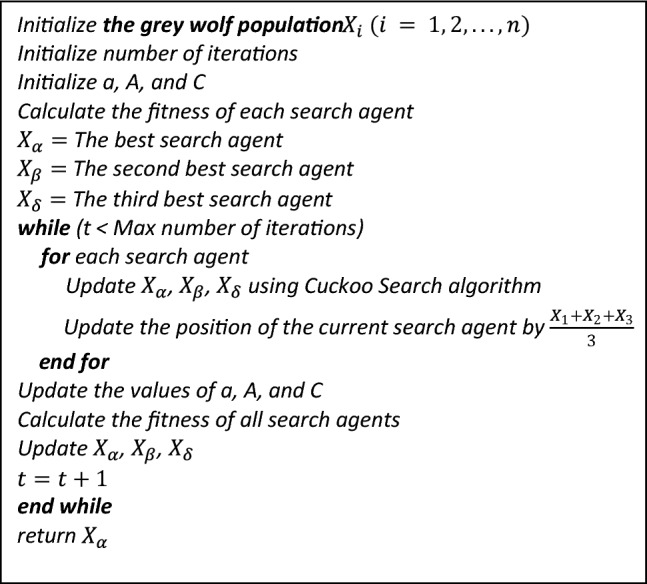
Pseudo code of GWOCS

So, the powerful ability of CS is used to improve the performance of GWO as CS updates the position of current search agent where after the calculation of $\vec{X}_{1} , \vec{X}_{2} , \vec{X}_{3}$ (the three best candidate solution) instead of directly using the mean value of the three parameters to update the position of current search agent, the values of $\vec{X}_{1} , \vec{X}_{2} , \vec{X}_{3}$ are first updated using Cuckoo search algorithm and then the position of the grey wolves is modified by $\frac{{\vec{X}_{1} + \vec{X}_{2} + \vec{X}_{3} }}{3}$. The pseudo code for the GWOCS is shown in Fig. 14.

In the optimization process, the fitness value of each search agent is determined by an objective function. This function is considered to be the most important factor in acquiring the required controller parameters. The objective function can be minimized or maximized to obtain the controller's intended response.

### Stability analysis

In this section stability analysis of IT2FLC for regulation of BGL for T1DP using Lyapunov function [60] is performed to determine the stability region of nonlinear system.

Our target is to design the control signal *U* such that the BGL of T1DP *x*_1_ follows the set BGL *R*, that is the error signal *e* → 0 as *t* → ∞ where:

16 $$e = R - x_{1}$$

So our lyapunov function candidate is chosen as

17 $$V\left( {e,\dot{e}} \right) = \frac{1}{2}\left( {e^{2} + \dot{e}^{2} } \right)$$

According to lyapunov stability theory for the system to be asymptotic stable, $\dot{V}\left( {e,\dot{e}} \right)$ must be negative definite [60].

By taking time derivative of Eq. (17), it yields:

18 $$\dot{V}\left( {e,\dot{e}} \right) = e\dot{e} + \dot{e}\mathop e\limits$$

By taking the time derivative of Eq. (16), it yields:

19 $$\dot{e} = - \mathop {x_{1} }\limits$$

By substitution for $\dot{x}_{1}$ from Eq. (1) in Eq. (19), it yields:

20 $$\dot{e} = p_{1} \left( {x_{1} - G_{b} } \right) + x_{1} x_{2} - x_{4}$$

By taking the second time derivative of Eq. (20), it yields:

21 $$\ddot{e} = p_{1} \dot{x}_{1} + x_{1} \dot{x}_{2} + \dot{x}_{1} x_{2} - \dot{x}_{4}$$

By substitution for $\dot{x}_{2} ,\dot{x}_{4}$ from Eqs. (2), (4) respectively in Eq. (21), it yields:

$$\ddot{e} = p_{1} \dot{x}_{1} + x_{1} \left( { - p_{2} x_{2} + p_{3} x_{3} - p_{3} I_{b} } \right) + \dot{x}_{1} x_{2} + p_{5} x_{4}$$

22 $$\ddot{e} = p_{1} \dot{x}_{1} - p_{2} x_{1} x_{2} + p_{3} x_{1} x_{3} - p_{3} x_{1} I_{b} + \dot{x}_{1} x_{2} + p_{5} x_{4}$$

By taking the third time derivative of Eq. (22), it yields:

23 $$\begin{aligned} e^{ \ldots } & = p_{1} \dot{x}_{1} - p_{2} x_{1} \dot{x}_{2} - p_{2} \dot{x}_{1} x_{2} + p_{3} x_{1} \dot{x}_{3} + p_{3} \dot{x}_{1} x_{3} \\ & \quad - p_{3} I_{b} \dot{x}_{1} + \dot{x}_{1} \dot{x}_{2} + \dot{x}_{1} x_{2} + p_{5} \dot{x}_{4} \\ \end{aligned}$$

By substitution for $\dot{x}_{3}$ from Eq. (3) in Eq. (23), it yields:

24 $$\begin{aligned} e^{ \ldots } & = \ddot{x}_{1} \left( {p_{1} + x_{2} } \right) + \dot{x}_{1} \left( {\dot{x}_{2} + p_{3} x_{3} - p_{3} I_{b} - p_{2} x_{2} } \right) \\ & \quad - p_{2} x_{1} \dot{x}_{2} + p_{3} x_{1} \left( { - p_{4} x_{3} + p_{4} I_{b} + u\left( t \right)} \right) + p_{5} \dot{x}_{4} \\ e^{ \ldots } & = \dot{x}_{1} \left( {p_{1} + x_{2} } \right) + \dot{x}_{1} \left( {\dot{x}_{2} + p_{3} x_{3} - p_{3} I_{b} - p_{2} x_{2} } \right) \\ & \quad - p_{2} x_{1} \dot{x}_{2} + p_{3} p_{4} I_{b} x_{1} - p_{3} p_{4} x_{1} x_{3} + p_{3} x_{1} u\left( t \right) + p_{5} \dot{x}_{4} \\ \end{aligned}$$

25 $$e^{ \ldots } = W\left( t \right) + p_{3} x_{1} u\left( t \right)$$

where:

$$\begin{aligned} W\left( t \right) & = \ddot{x}_{1} \left( {p_{1} + x_{2} } \right) + \dot{x}_{1} \left( {\dot{x}_{2} + p_{3} x_{3} - p_{3} I_{b} - p_{2} x_{2} } \right) \\ & \quad - p_{2} x_{1} \dot{x}_{2} + p_{3} p_{4} I_{b} x_{1} - p_{3} p_{4} x_{1} x_{3} + p_{5} \dot{x}_{4} \\ \end{aligned}$$

Hence, we want to verify that $\dot{V}\left( {e,\dot{e}} \right) < 0$, then from Eq. (18):

26 $$e\dot{e} + \dot{e}\ddot{e} < 0$$

When we analyze Eq. (26), it can be achieved within the following four cases:

Case 1:

If *e* and $\dot{e}$ are both positive sign, it requires that:$\ddot{e} < - e$.

By taking the third time derivative of Eq. (26), it yields:

27 $$e^{ \cdots } < - \dot{e}$$

By substitution the value of $\dot{e},e^{ \ldots }$ from Eqs. (19), (25) respectively in Eq. (27), it yields:

28 $$W\left( t \right) + p_{3} x_{1} u\left( t \right) < \dot{x}_{1}$$

By Solving for *u*(*t*) from Eq. (28), it yields:

29 $$u\left( t \right) < \frac{{\dot{x}_{1} - W\left( t \right)}}{{p_{3} x_{1} }}$$

Case 2:

If *e* and $\dot{e}$ are both negative sign, it requires that:

30 $$\ddot{e} > e$$

By taking the third time derivative of Eq. (30), it yields:

31 $$e^{ \ldots } > \dot{e}$$

By substitution the value of $\dot{e},e^{ \ldots }$ from Eqs. (19), (25) respectively in Eq. (31), it yields:

32 $$W\left( t \right) + p_{3} x_{1} u\left( t \right) > - \dot{x}_{1}$$

By Solving for *u*(*t*) from Eq. (32), it yields:

33 $$u\left( t \right) > \frac{{ - \dot{x}_{1} - W\left( t \right)}}{{p_{3} x_{1} }}$$

Case 3:

If *e* is positive sign and $\dot{e}$ is a negative sign, it requires that:

34 $$\ddot{e} > - e$$

By taking the third time derivative of Eq. (34), it yields:

35 $$e^{ \ldots } > - \dot{e}$$

By substitution the value of $\dot{e},e^{ \ldots }$ from Eqs. (19), (25) respectively in Eq. (35), it yields:

36 $$W\left( t \right) + p_{3} x_{1} u\left( t \right) > \dot{x}_{1}$$

By Solving for *u*(*t*) from Eq. (36), it yields:

37 $$u\left( t \right) > \frac{{\dot{x}_{1} - W\left( t \right)}}{{p_{3} x_{1} }}$$

Case 4:

If *e* is negative sign and $\dot{e}$ is a positive sign, it requires that:

38 $$\ddot{e} < e$$

By taking the third time derivative of Eq. (38), it yields:

39 $$e^{ \ldots } < \dot{e}$$

By substitution the value of $\dot{e},e^{ \ldots }$ from Eqs. (19), (25) respectively in Eq. (39), it yields:

40 $$W\left( t \right) + p_{3} x_{1} u\left( t \right) < - \dot{x}_{1}$$

By Solving for *u*(*t*) from Eq. (40), it yields:

41 $$u\left( t \right) < \frac{{ - \dot{x}_{1} - W\left( t \right)}}{{p_{3} x_{1} }}$$

Consequently, the constraints in Eqs. (29), (33), (37), and (41) for the control signal *U* ensure the asymptotic stability of the system and determine the stability region.

## Results and discussion

The main objective of the proposed controller IT2FLC is to maintain BGL for a T1DP within standard-safe range between 70 and 180 mg/dl even the presence of parametric uncertainty of the model (the parameters alters from one patient to other), uncertain meal disturbance, and physical exercise that can burn glucose. That is mean bringing BGL less than 180 mg/dl within almost 120 to 150 min after meals are provided with avoiding hypoglycemia situation (BGL < 50 mg/dl) at any instance. Consequently in this section, simulation results of IT2FLC using GWOCS for regulation of BGL for EBMM are proposed in six different scenarios using matlab functions for IT2FLS in Matlab/simulink platform [61, 62].

The nominal values and range of parameters for the EEBM model used in simulation are shown in Table 3 [33].

**Table 3:** Nominal value and range of parameters for EBMM

parameters	Nominal value	Range
*p*_1_	1 × 10^−7^	[0.8 × 10^−7^, 1.2 × 10^−7^]
*p*_2_	0.015	[0.0105, 0.0195]
*p*_3_	2 × 10^−6^	[1.4 × 10^−6^, 2.6 × 10^−6^]
*p*_4_	0.2	[0.14, 0.26]
*p*_5_	0.05	[0.04, 0.06]

Scenario 1:

The main objective of this scenario is to investigate the behavior of IT2FLC for nominal parameters of EBMM under difficult conditions that at the start of simulation: T1DP is in the state of hyperglycemia (BGL > 180 mg/dl) moreover a large meal containing high amount of glucose is provided to T1DP. Configuration of this scenario is: simulation period is set to 800 min, the initial conditions at *t* = 0 min of the state variables for EBMM are *x*_1_ = 250 mg/dl reflects that T1DP is already in the state of hyperglycemia at the start of simulation, *x*_2_ = 0 min^−1^, *x*_3_ = 7 mU/l ensuring that there is no insulin infusion before the starting of simulation, *x*_4_ = 10 mg/dl/min means that a large meal disturbance with a very high glucose content is provided at the start of the simulation. The basal values for glucose and insulin level are *G*_*b*_ = 80 mg/dl, *I*_*b*_ = 7 mU/l respectively. And the nominal values for parameters *p*_1_, *p*_2_, *p*_3_, *p*_4_, and *p*_5_ for EBMM model are chosen from Table 3.

**Fig. 15:**
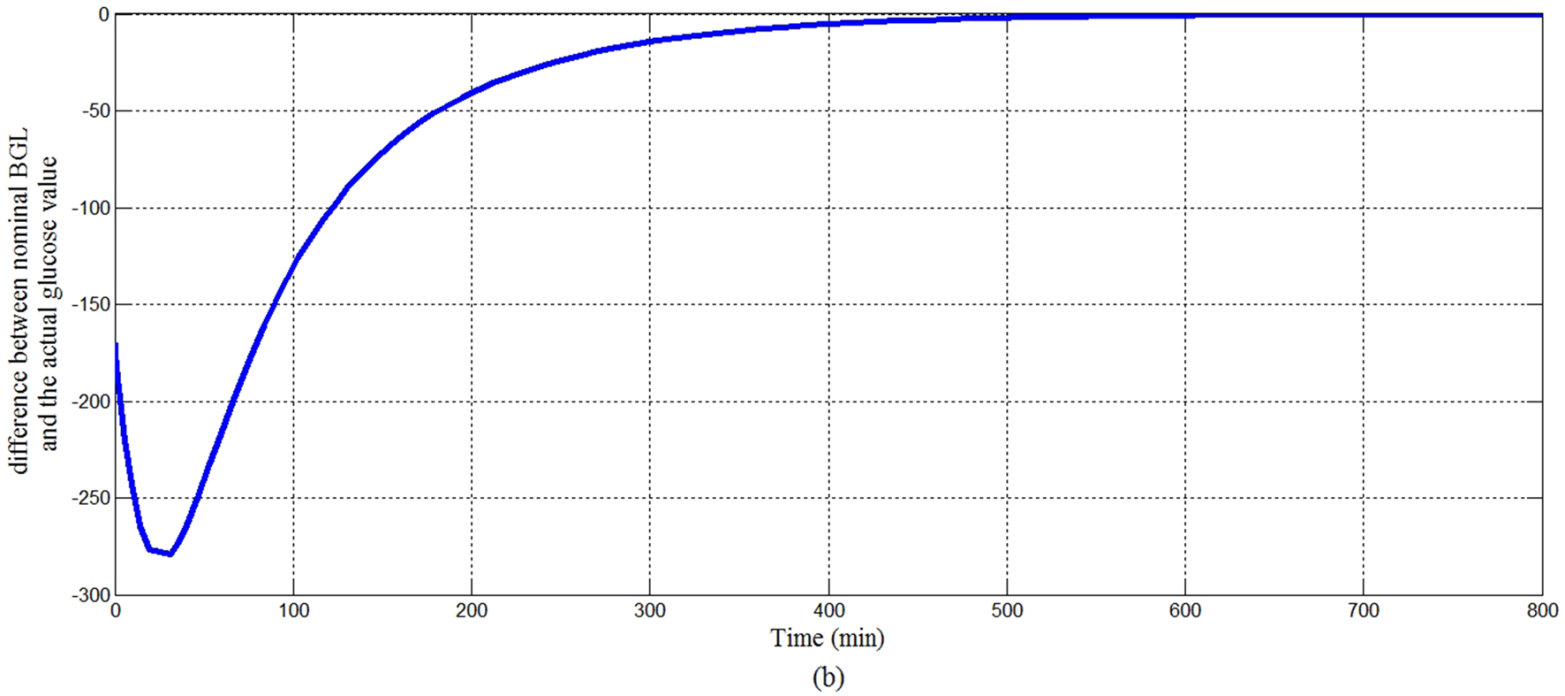
The difference between nominal BGL and the actual glucose value

**Fig. 16:**
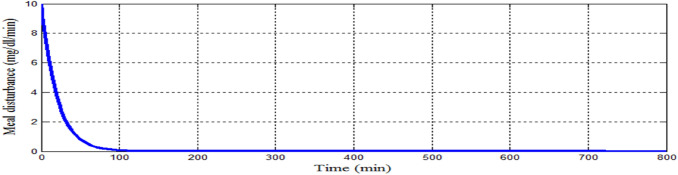
Meal disturbance for scenario 1

**Fig. 17:**
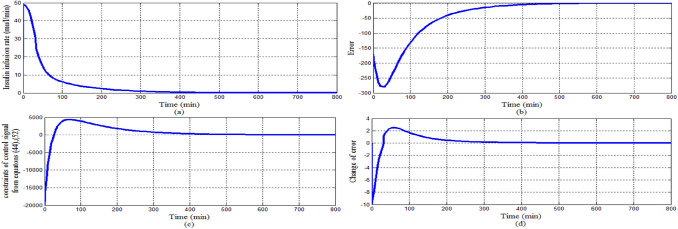
Stability analysis for scenario 1

Figure 15 showed that the proposed controller lowered BGL more than 180 mg/dl from the state of hyperglycemia within 120 min in spite of a meal disturbance with a very high glucose content was provided at the start of the simulation, in addition BGL stabilized at basal glucose level of 80 mg/dl without any hypoglycemia at any instance as desired. The shape of the meal disturbance is shown in Fig. 16. As shown in Fig. 17a response of insulin infusion rate is always positive and smooth during the all simulation period it reflects that the proposed controller is simple, not complicated doesn't need any excessive method or algorithm to inject glucagon or glucose to avoid hypoglycemia.

For investigating the asymptotic stability of the system Fig. 17a shows that the control signal follows the constraints of Eqs. (33), (41) proved in Sect. 2 that guarantee the stability of the system. Within the time interval in which error and change of error signal are both negative sign as shown in Fig. 17b, d respectively, the control signal follows Eq. (33) that insulin infusion rate is larger than the right hand side of Eq. (33) as shown in Fig. 17c. Whereas within the time interval that error signal is negative sign and change of error signal is a positive sign, the control signal follows Eq. (41) that insulin infusion rate is less than the right hand side of Eq. (41).

Scenario 2:

In this scenario simulation period is set to 1440 min to investigate the behavior of the proposed controller during 24 h of one T1DP’s daily life. Three different meals 5 mg/dl/min for breakfast, 8 mg/dl/min for lunch, and 8 mg/dl/min dinner are provided to a T1DP at 480 min (8 am), 720 min (12 am), and 1200 min (8 am) respectively. Nominal values of parameters *p*_1_, *p*_2_, *p*_3_, *p*_4_, and *p*_5_ for EBMM model are chosen from Table 3 and the initial conditions of the state variables for EBMM at the start of simulation are *x*_1_ = *G*_*b*_ = 80 mg/dl, *x*_2_ = 0 min^−1^, *x*_3_ = *I*_*b*_ = 7 mU/l, and *x*_4_ = 0 mg/dl/min (T1DP is in fasting condition before starting of simulation).

**Fig. 18:**
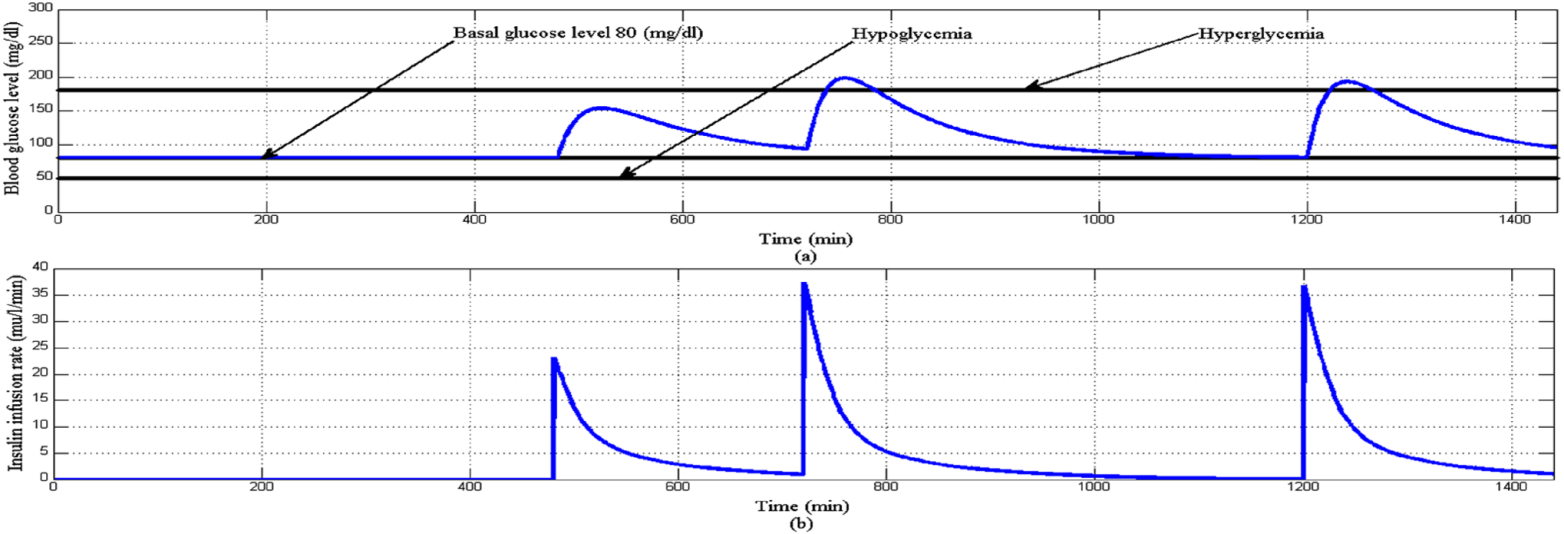
**a** BGL response for scenario 2 **b** insulin infusion rate for scenario 2

Initially at the start of simulation when the BGL is at basal glucose level as shown in Fig. 18a there is no insulin infusion as shown in Fig. 18b, after each of the three meals are provided to T1DP separately at its time BGL increases proportionally according to the amount of glucose content in each of the three meals. And dependency on the above insulin infusion rate is increased proportionally to any changes in BGL forcing it to stabilize around the basal glucose level avoiding both hyperglycemia and hypoglycemia situation as shown in Fig. 18a.

**Fig. 19:**
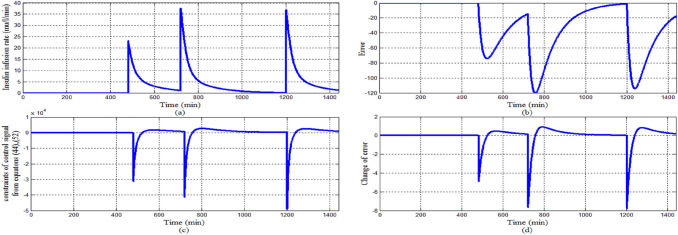
Stability analysis for scenario 2

Also in this scenario in order to investigating the asymptotic stability of the system Fig. 19a shows that the control signal follows the constraints of Eqs. (33), (41) that guarantee the stability of the system. Within the time interval in which error and change of error signal are both negative sign as shown in Fig. 19b, d respectively, the control signal follows Eq. (33) that insulin infusion rate is larger than the right hand side of Eq. (33) as shown in Fig. 19c. Whereas within the time interval that error signal is negative sign and change of error signal is a positive sign, the control signal follows Eq. (41) that insulin infusion rate is less than the right hand side of Eq. (41).

Scenario 3:

In this scenario 100 simulation rounds are run to study the behavior 100 virtual-different T1DPs (parametric uncertainty of EBMM) along with a large meal disturbance. At the start of each simulation round the parameters *p*_1_, *p*_1_, *p*_3_, *p*_4_, and *p*_5_ are randomly chosen from Table 3 with variations ± 20% to constitute a 100 virtual T1DPs. Configuration of this scenario is: simulation period is set to 800 min, the initial conditions of the state variables for EBMM at the start of each simulation round are *x*_1_ = 250 mg/dl, *x*_2_ = 0 min^−1^, *x*_3_ = 7 mU/l, and *x*_4_ = 10 mg/dl/min. The basal values for glucose and insulin level are *G*_*b*_ = 80 mg/dl, *I*_*b*_ = 7 mU/l respectively.

**Fig. 20:**
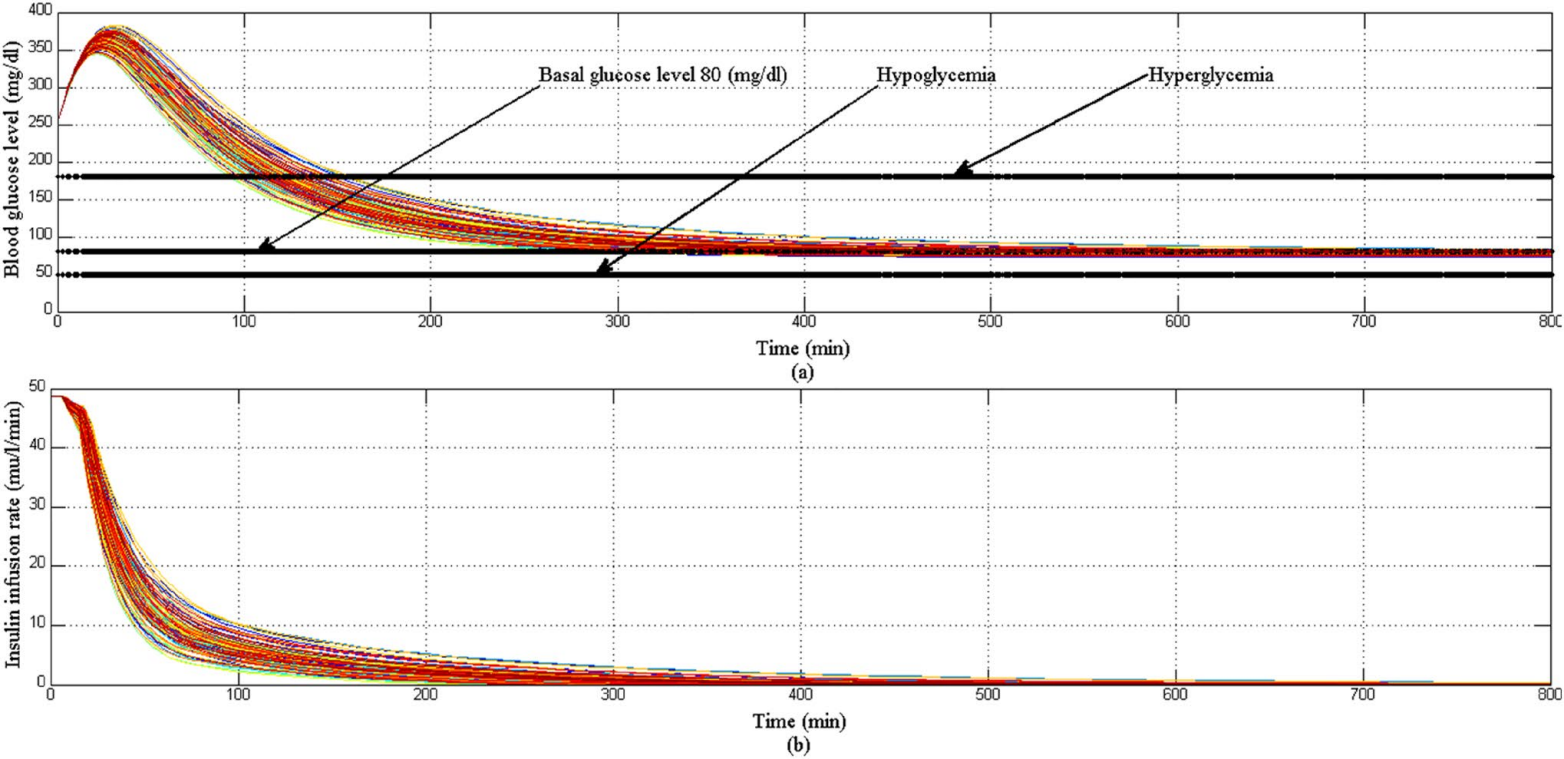
**a** BGL response for Scenario 3 **b** insulin infusion rate for Scenario 3

Figure 20a shows that despite the presence of parametric uncertainty of EBMM along with a large meal disturbance, the BGL has stabilized near the basal glucose level for all 100 virtual T1DPs within standard-safe range avoiding the situation of hyperglycemia moreover avoiding hypoglycemia situation at any instance. Also insulin infusion rate is still positive and smooth as shown in Fig. 20b.

Scenario 4:

In this scenario 100 simulation rounds are run to study the behavior of varying both meal disturbance and its time randomly on a 100 virtual-different T1DPs. So a random amount of meal disturbance is set to vary from 5 to 8 mg/dl/min along with a random meal time varies from 0 to 60 min.

Configuration of this scenario is: simulation period is set to 800 min, the initial conditions of the state variables for EBMM at the start of each simulation round are *x*_1_ = *G*_*b*_ = 80 mg/dl, *x*_2_ = 0 min^−1^,* x*_3_ = *I*_*b*_ = 7 mU/l, and *x*_4_ = 0 mg/dl/min (all 100 virtual T1DPs are in fasting condition), the basal values for glucose and insulin level are *G*_*b*_ = 80 mg/dl, *I*_*b*_ = 7 mU/l respectively, and the parameters *p*_1_, *p*_2_, *p*_3_, *p*_4_, and *p*_5_ are randomly chosen from Table 3 with variations ± 20% to constitute a 100 virtual T1DPs.

**Fig. 21:**
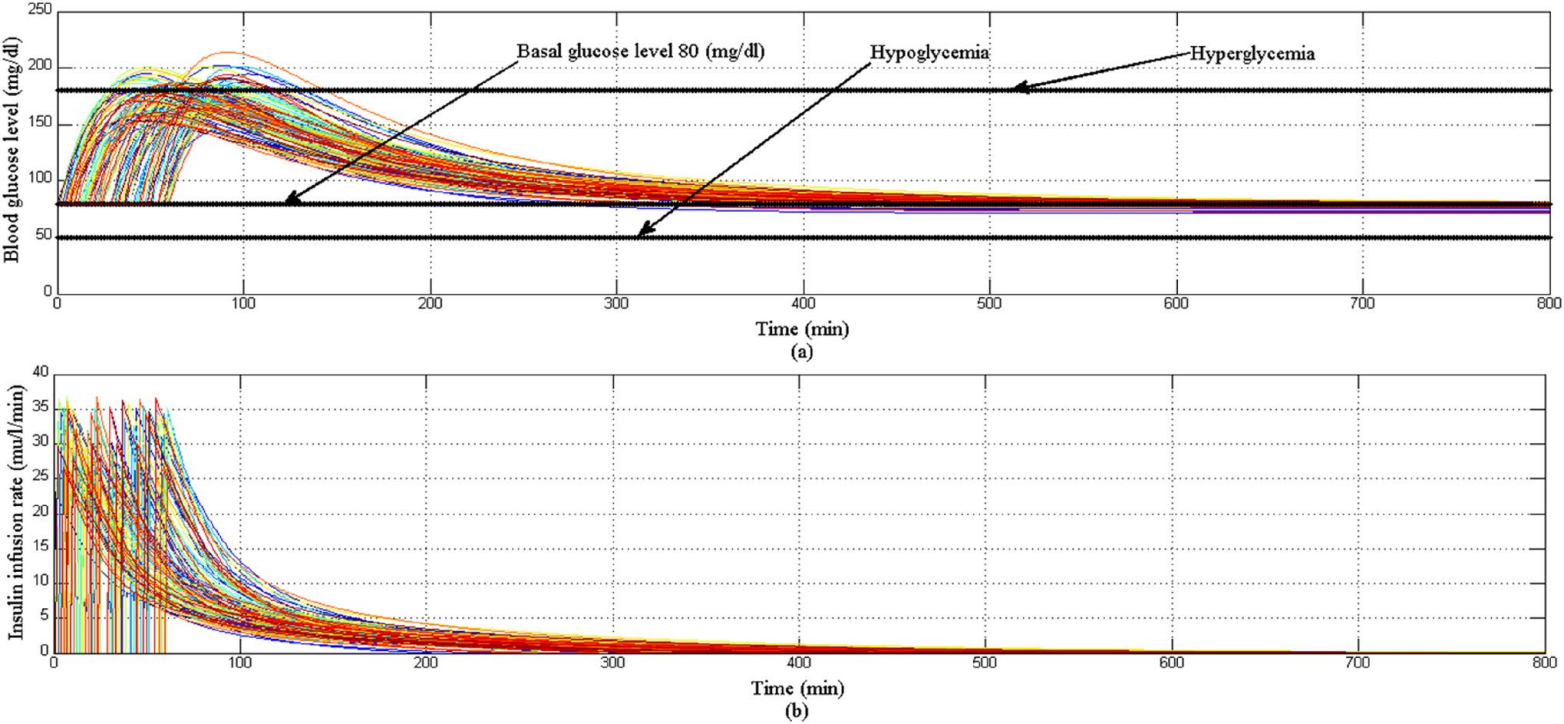
**a** BGL response for scenario 4 **b** insulin infusion rate for scenario 4

Figure 21a shows that despite the parametric uncertainty of EBMM along with uncertain both meal disturbance and meal time, the proposed controller forces BGL to stabilize around the basal glucose level avoiding hyperglycemia situation and avoiding hypoglycemia situation at any instance. Figure 21b shows the insulin infusion rate for this scenario.

**Fig. 22:**
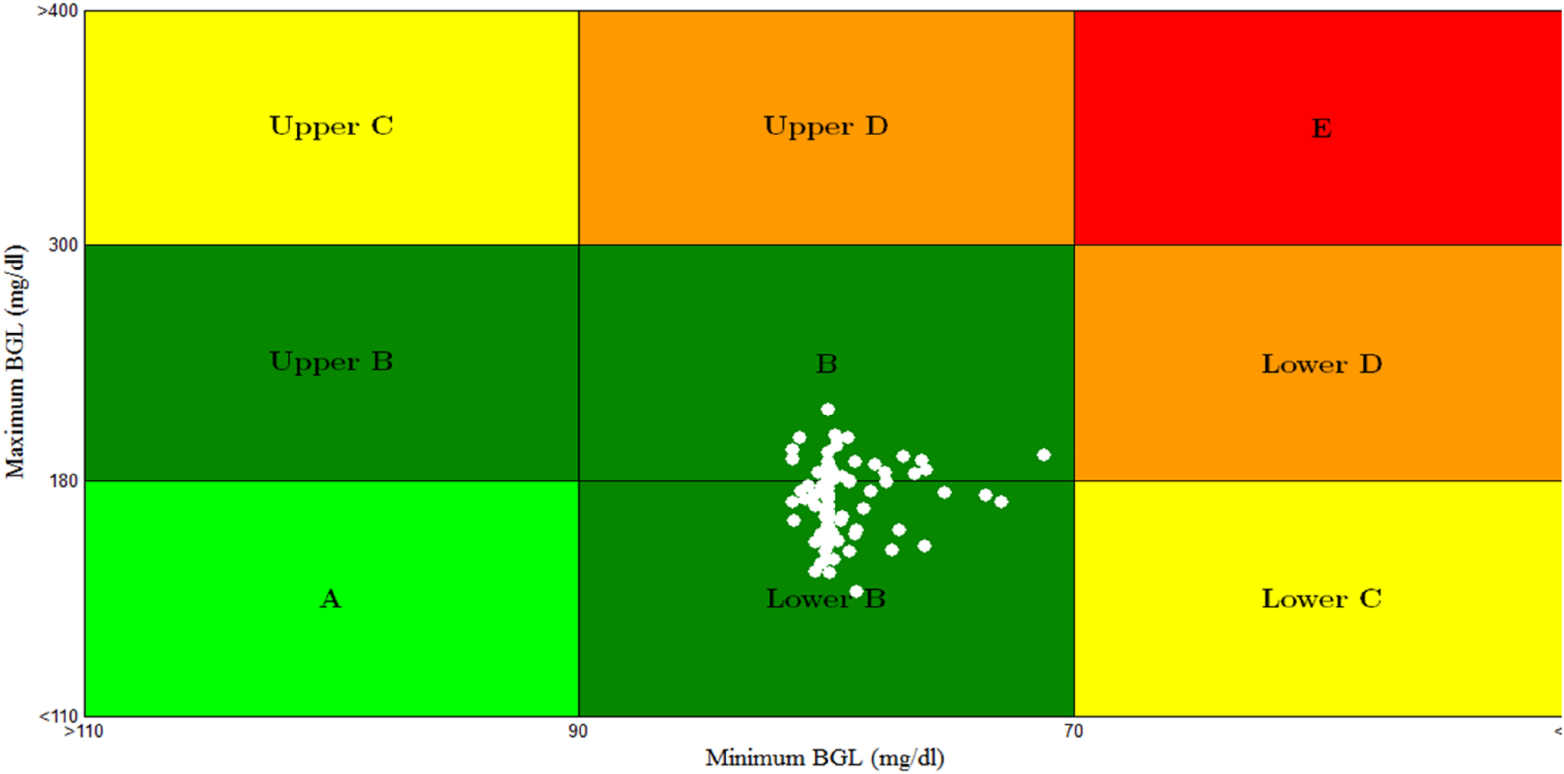
CVGA plot for scenario 4

Also the result of CVGA for scenario 4 of 100 T1DPs is shown in Fig. 22. CVGA is a graphical tool used to measure the quality of the closed loop control system in regulating the BGL for the population of T1DPs by expressing the minimum/maximum glucose values for each of T1DPs [48]. Where each of T1DP in CVGA plot is expressed by a white dot, X-axis and Y-axis refers to minimum and maximum BGL within the simulation period respectively.

Figure 22 displays that all 100 white dots are confined to the green safe regions; grid B and grid lower B. It reflects that the time period spent by 100 T1DPs in hyperglycemia is minimized in spite of uncertain both meal disturbance and meal time, and also the minimum glucose values for all the 100 T1DPs are within safe range 70–90 mg/dl ensuring that hypoglycemia is avoided.

Scenario 5:

In this scenario a 200 virtual T1DPs are used with 1440 min simulation period to simulate a more realistic situation for T1DPs. Consequently the objective of this scenario is to investigate the behavior of the proposed controller within 24 h of T1DP’s daily life, so a three variable different meals for breakfast, lunch, and dinner are randomly chosen from the interval {4, 9)} mg/dl/min are provided to each of the 200 virtual T1DPs at 480 min (8 am), 720 min (12 am), and 1200 min (8 am) respectively. At start of each simulation period the parameters *p*_1_, *p*_2_, *p*_3_, *p*_4_, and *p*_5_ are randomly chosen from Table 3 with variations ± 20% to constitute a 200 virtual T1DPs. The initial conditions of the state variables are *x*_1_ = *G*_*b*_ = 80 mg/dl, *x*_2_ = 0 min^−1^, *x*_3_ = *I*_*b*_ = 7 mU/l, *x*_4_ = 0 mg/dl/min (all 200 virtual T1DPs are in fasting condition before starting of simulation), and the basal values for glucose and insulin level are *G*_*b*_ = 80 mg/dl, *I*_*b*_ = 7 mU/l respectively.

**Fig. 23:**
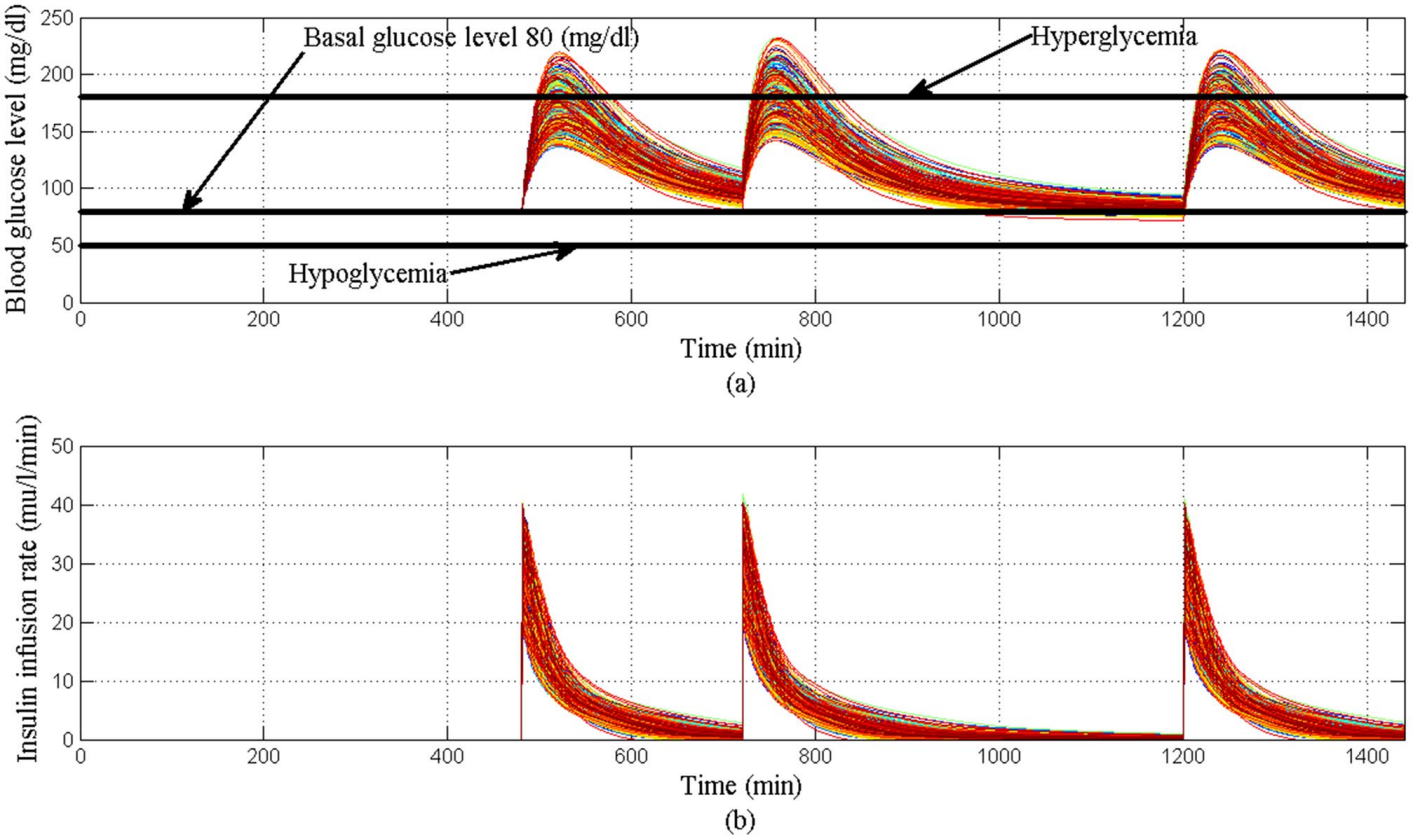
**a** BGL response for scenario 5 **b** insulin infusion rate for scenario 5

Initially at the start of simulation when the BGL of all T1DPs is at basal glucose level as shown in Fig. 23a there is no insulin infusion as shown in Fig. 23b, after each of the three meals are provided to each of all T1DPs separately at its time, BGL increases proportionally according to the amount of glucose content in each of the three meals. And dependency on the above, insulin infusion rate is increased proportionally to any changes in BGL forcing it to stabilize around the basal glucose level avoiding both hyperglycemia and hypoglycemia situation as shown in Fig. 23a.

Despite the parametric uncertainty of EBMM, and the different three meal disturbances, the proposed controller forces BGL to stabilize around the basal glucose level, and both hyperglycemia and hypoglycemia situations were still avoided.

**Fig. 24:**
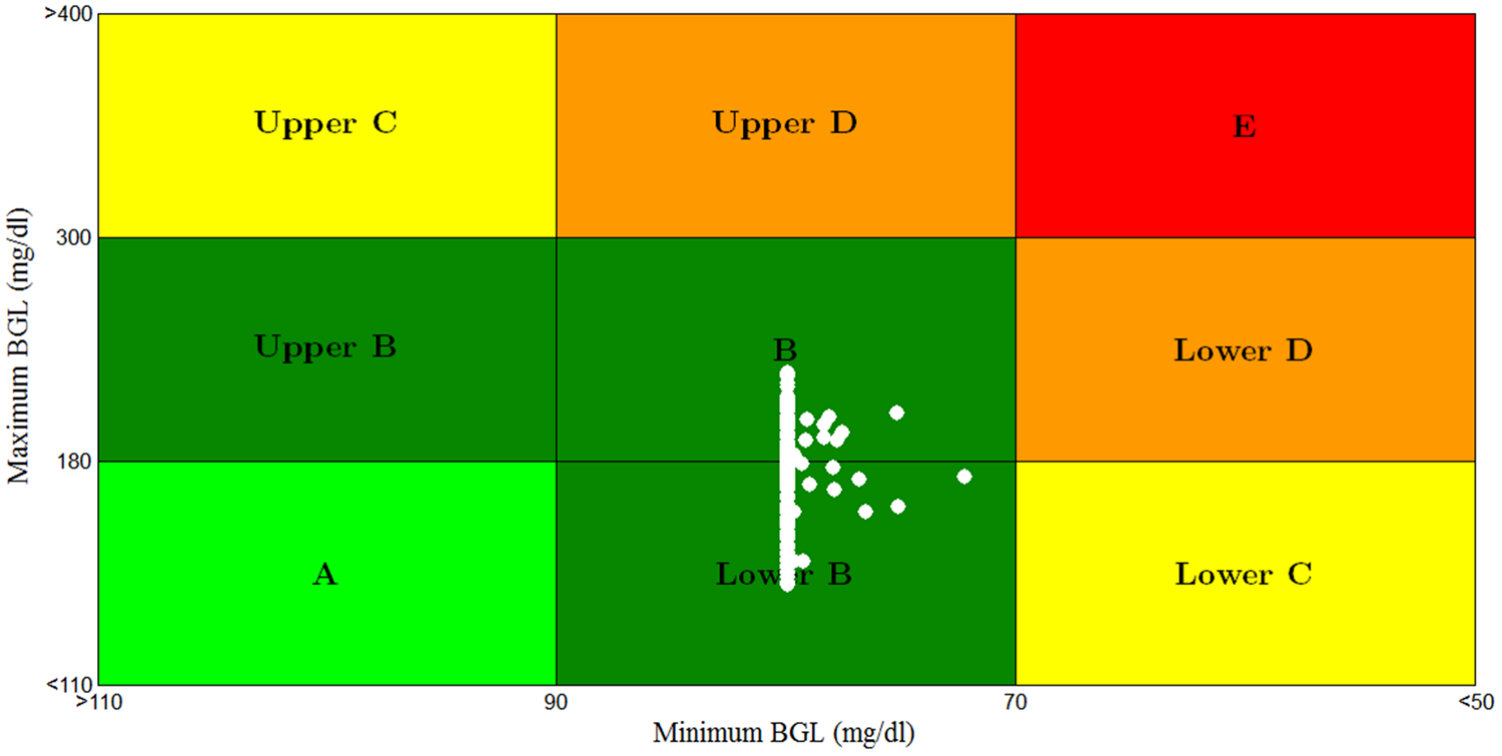
CVGA plot for scenario 5

The result of CVGA for scenario 5 is shown in Fig. 24.

**Fig. 25:**
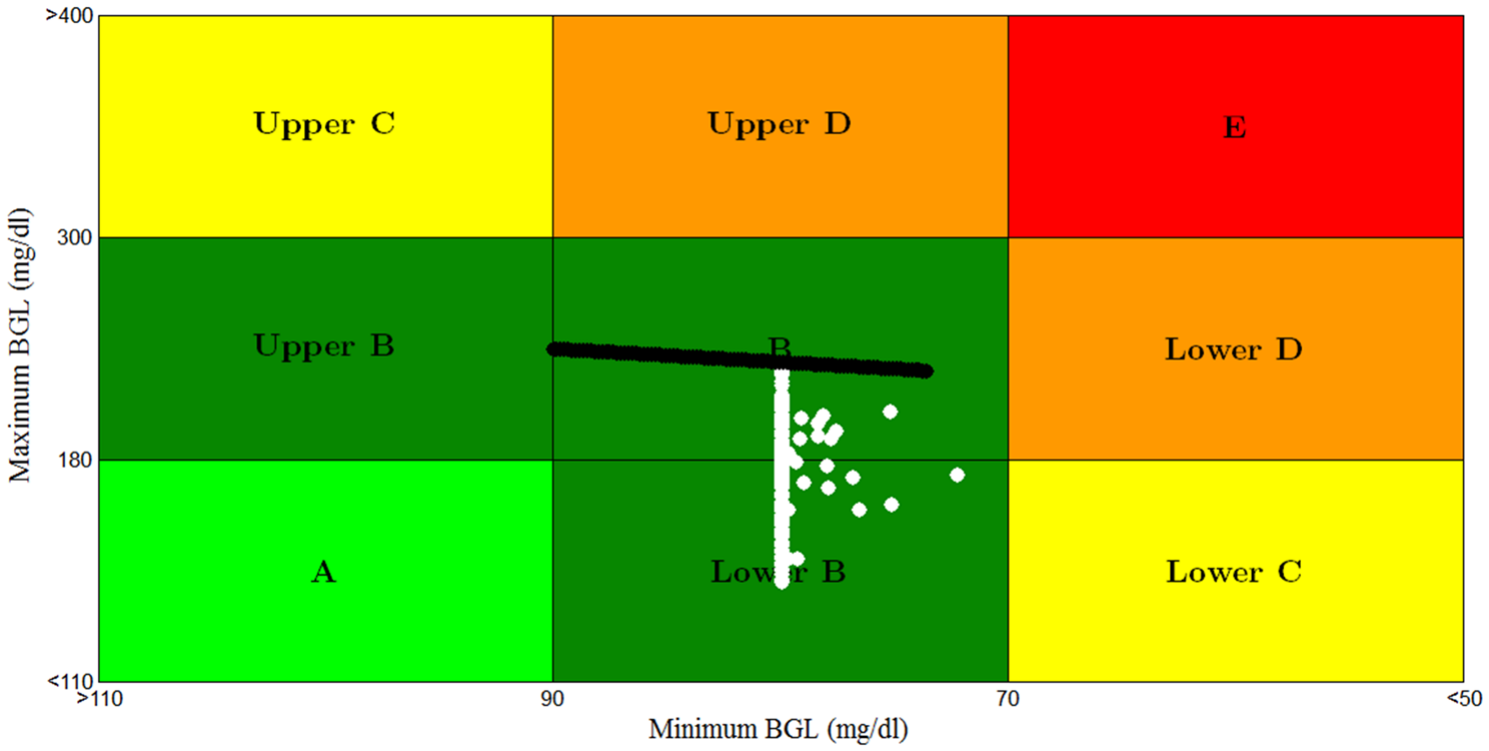
Comparison between CVGA plot of the proposed scenario 5 (the white circles) and CVGA plot for scenario 3 of work presented in [33] (the black circles)

For the sake of comparison to other work Fig. 25 displays the results of CVGA plot for scenario 3 of work presented in [33] (the black circles) and CVGA plot of the proposed scenario 5 (the white circles). For a fair comparison, all parameters and configuration of scenario 5 were selected to be identical to scenario 3 of work presented in [33].

From Fig. 25, the lowest glucose levels for the proposed controller are between 70 and 90 mg/dl, matching those of CVGA plot in [33]. The maximum glucose values for the proposed controller are between 141.374 and 227.58 mg/dl, whereas maximum glucose values are almost between 228 and 240 mg/dl of [33]. 89.5% of the 200 T1DPs for the proposed technique in Fig. 25 are almost centered at the basal glucose level 80 mg/dl and the rest are between 72.3 and 80 mg/d. whereas 100% of the T1DPs are distributed horizontally between 73.6 and 90 mg/dl in [33]as shown in Fig. 25.

Stabilizing the blood glucose level close to the basal glucose level without the occurrence of hyperglycemia or hypoglycemia circumstances is regarded as an important improvement.

**Fig. 26:**
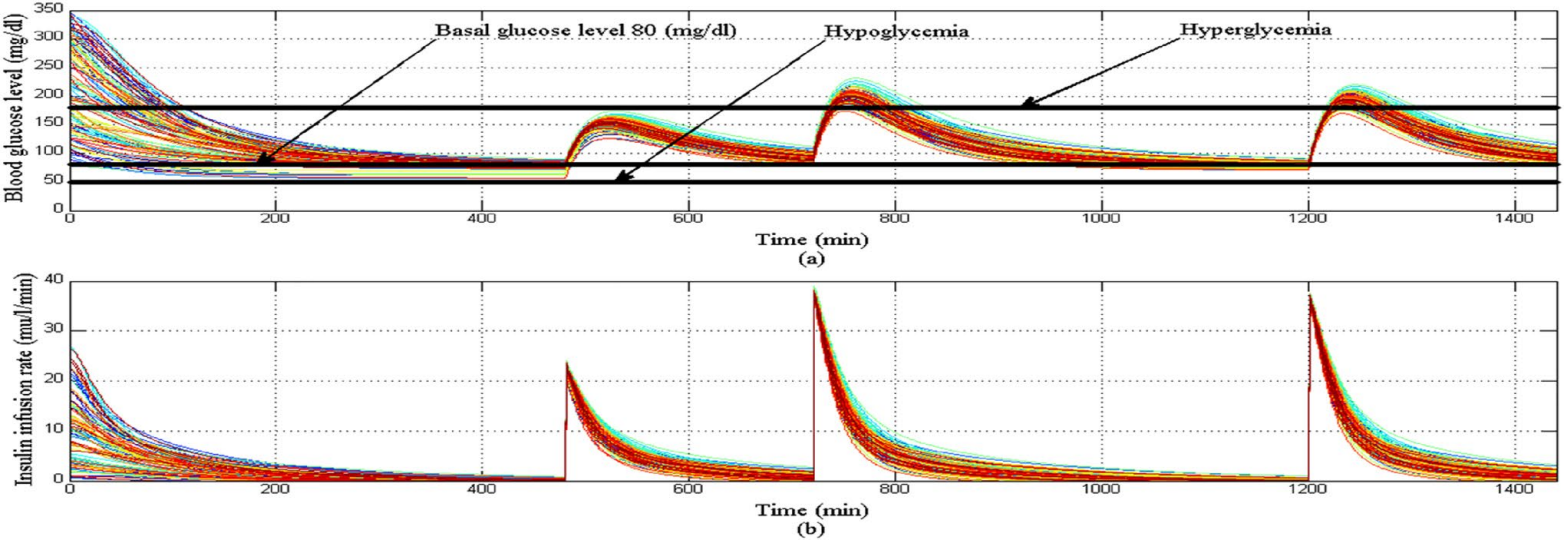
**a** BGL response for scenario 6 **b** insulin infusion rate for scenario 6

Scenario 6:

Other critical scenario is presented here with the same configuration as scenario 5 moreover a random initial conditions of the state variables for EBMM is considered. At start of each simulation round *x*_1_ varies randomly form 80 to 350 mg/dl, *x*_2_ varies randomly form 0 to 0.01 min^−1^, and *x*_3_ varies randomly form 0 to 20 mU/l, also the parameters *p*_1_, *p*_2_, *p*_3_, *p*_4_, and *p*_5_ are still randomly chosen from Table 3 with variations ± 20% to constitute a 100 virtual T1DPs. Initially at the start of simulation when the BGL is above the basal glucose level as shown in Fig. 26a, insulin infusion rate is increased proportionally to the value of BGL as shown in Fig. 26b forcing it to stabilize near the basal glucose level. BGL lowered below180 mg/dl within approximately at most 112 min for all T1DPs. Also after each of the three meals are provided to each of all T1DPs separately at its time BGL increases proportionally according to the amount of glucose content in each of the three meals. And dependency on the above insulin infusion rate is increased again forcing BGL again to stabilize around the basal glucose level. Despite all of the following: random initial conditions of the state variables for EBMM, parametric uncertainty, and different three meal disturbances the proposed controller forces BGL to stabilize around the basal glucose level. And both hyperglycemia and hypoglycemia situations were still avoided.

**Fig. 27:**
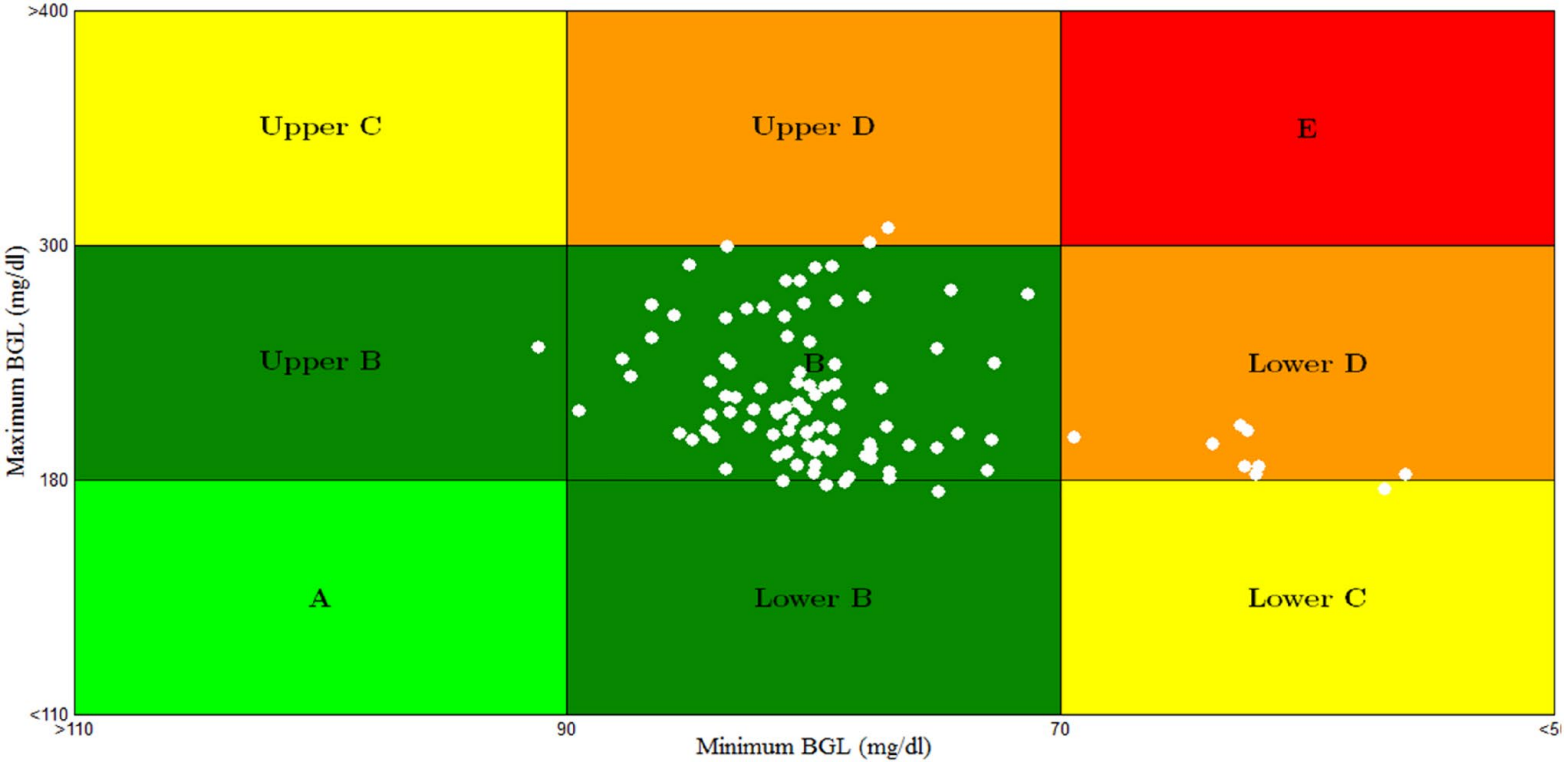
CVGA plot for scenario 6

Also the result of CVGA for scenario 6 is shown in Fig. 27. Due to the elevated values of BGL because of random initial conditions of the state variables at the start of simulation there are 9% of T1DPs are in the Lower D region and Lower C region, 2% are in upper D region, 1% are in upper B region. While 88% of T1DPs are confined to the green safe regions; grid B and grid lower B reflecting that the time period in hyperglycemia is minimized. In spite of the minimum glucose values for 9% of T1DPs are less than 70 mg/dl, hypoglycemia situation (BGL < 50 mg/dl) is still avoided.

**Fig. 28:**
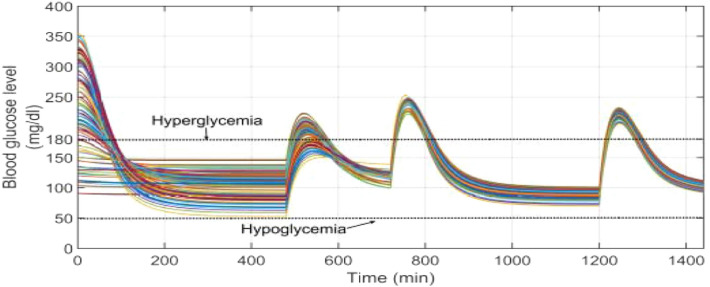
BGL response for scenario 4 of work presented in [33]

Figure 28 displays BGL response for scenario 4 of work presented in [33], all parameters and configuration of scenario 6 were selected to be identical to scenario 4 of work presented in [33]. By examining the two figures it is noted that despite the random initial conditions of states variable at the start of simulation, the proposed technique in Fig. 26a stabilized BGL within range from 50 to 89.5 mg/dl, whereas in Fig. 28 it is stabilized within range 50 to 150 mg/dl. After the breakfast meal is provided BGL increased in the two figures and then stabilized within range 80 to 112 mg/dl in Fig. 26a whereas in Fig. 28 BGL stabilized within range 100 to 140 mg/dl. Also after the lunch meal is provided BGL increased in the two figures and then stabilized within range 71 to 91 mg/dl in Fig. 26a Whereas in Fig. 28 stabilized within range 70 to 100 mg/dl. Finally after the dinner meal is provided BGL increased in the two figures and then stabilized within range 78 to 120 mg/dl in Fig. 26a whereas in Fig. 28 stabilized within range 90 to 110 mg/dl.

From above results, it is noted that the proposed technique will stabilize and regulate the BGL at a standard-safe level. The IT2FLC with the conjunction of GWOCS significantly decreases the effect of uncertainty and disturbances as shown in different scenarios in which T1DP can faces during his daily life.

**Fig. 29:**
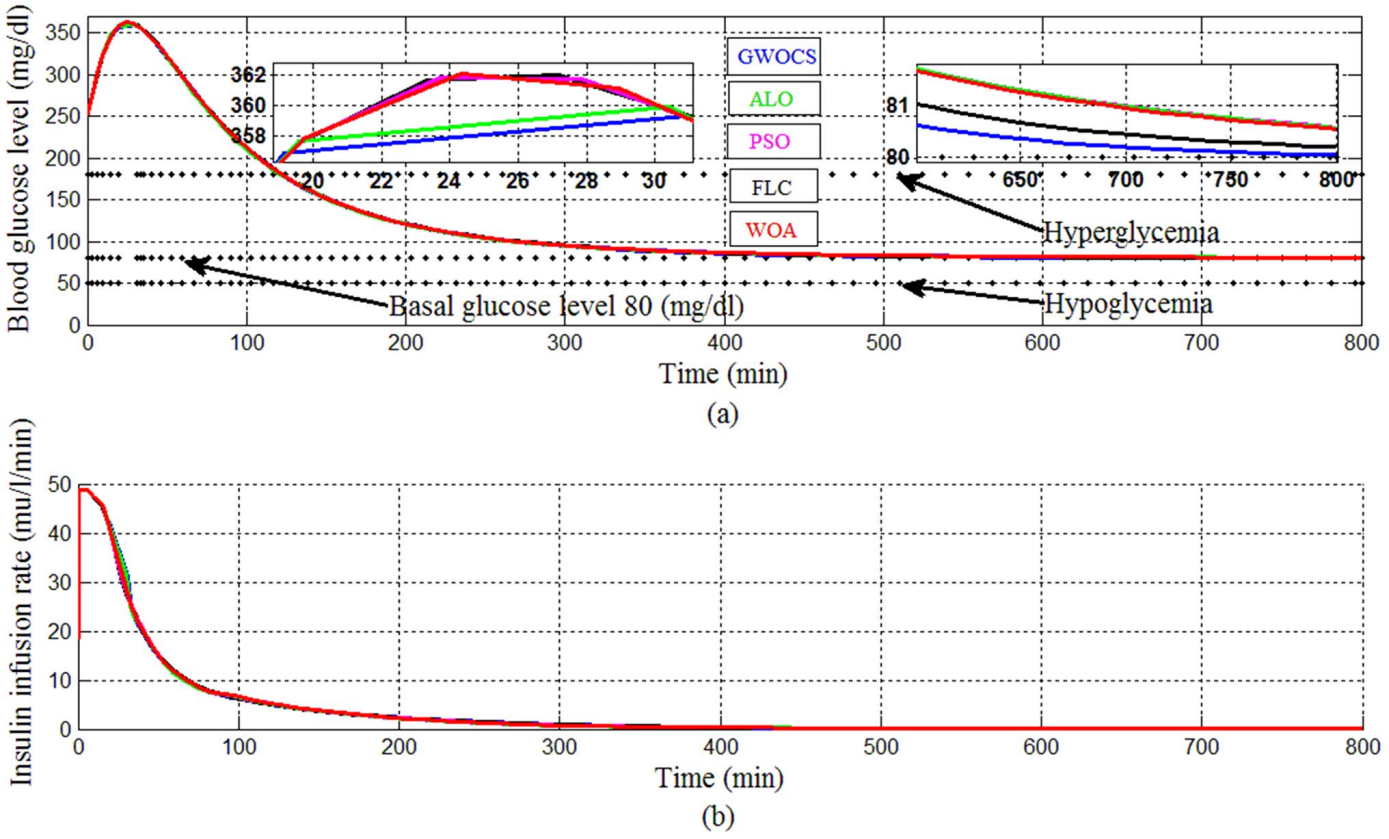
Comparison of GWOCS, ALO, PSO, WOA and FLC

Figure 29 shows a comparison includes IT2FLC using different optimization methods such as ALO, GWOCS, PSO, WOA and FLC method to infuse the proper amount of exogenous insulin. A difficult condition of EBMM is considered in this comparison, which is at the start of simulation: T1DP is in the state of hyperglycemia (BGL > 180 mg/dl) moreover a large meal containing high amount of glucose is provided to T1DP. simulation period is set to 800 min, the initial conditions at *t* = 0 min of the state variables for EBMM are *x*_1_ = 250 mg/dl reflects that T1DP is already in the state of hyperglycemia at the start of simulation, *x*_2_ = 0 min^−1^, *x*_3_ = 7 mU/l ensuring that there is no insulin infusion before the starting of simulation, *x*_4_ = 10 mg/dl/min means that a large meal disturbance with a very high glucose content is provided at the start of the simulation. The basal values for glucose and insulin level are *G*_*b*_ = 80 mg/dl, *I*_*b*_ = 7 mU/l respectively. And the nominal values for parameters *p*_1_, *p*_2_, *p*_3_, *p*_4_, and *p*_5_ for EBMM model are chosen from Table 3.

In order to make a clear comparison some performance criteria such: Rise Time, Settling Time, Percentage Overshoot, and MAE are used. Table 4 shows the values of the parameters, *wi*, *wi*1, *wi*2 and numerical results of performance criteria for the different optimization methods of IT2FLC and also for FLC method.

**Table 4:** Numerical results for the different optimization methods of IT2FLC and FLC

	Wi	Wi1	Wi2	MAE	Rise time	Settling time	Overshoot
IT2FLC-PSO	0.0592	0.0176	0.2608	77.8116	198.1273	418.7788	352.3602
FLC	0	0	0	78.3085	199.3745	406.4778	352.5704
IT2FLC-GWOCS	0.0033898	0.030848	0.28076	73.9780	196.2754	394.5724	349.1818
IT2FLC-ALO	0.059846	0.01766	0.060536	74.4466	197.4069	418.5425	349.8764
IT2FLC-WOA	0.055534	0.014968	0.089904	76.6695	198.2361	417.4637	352.6396

**Fig. 30:**
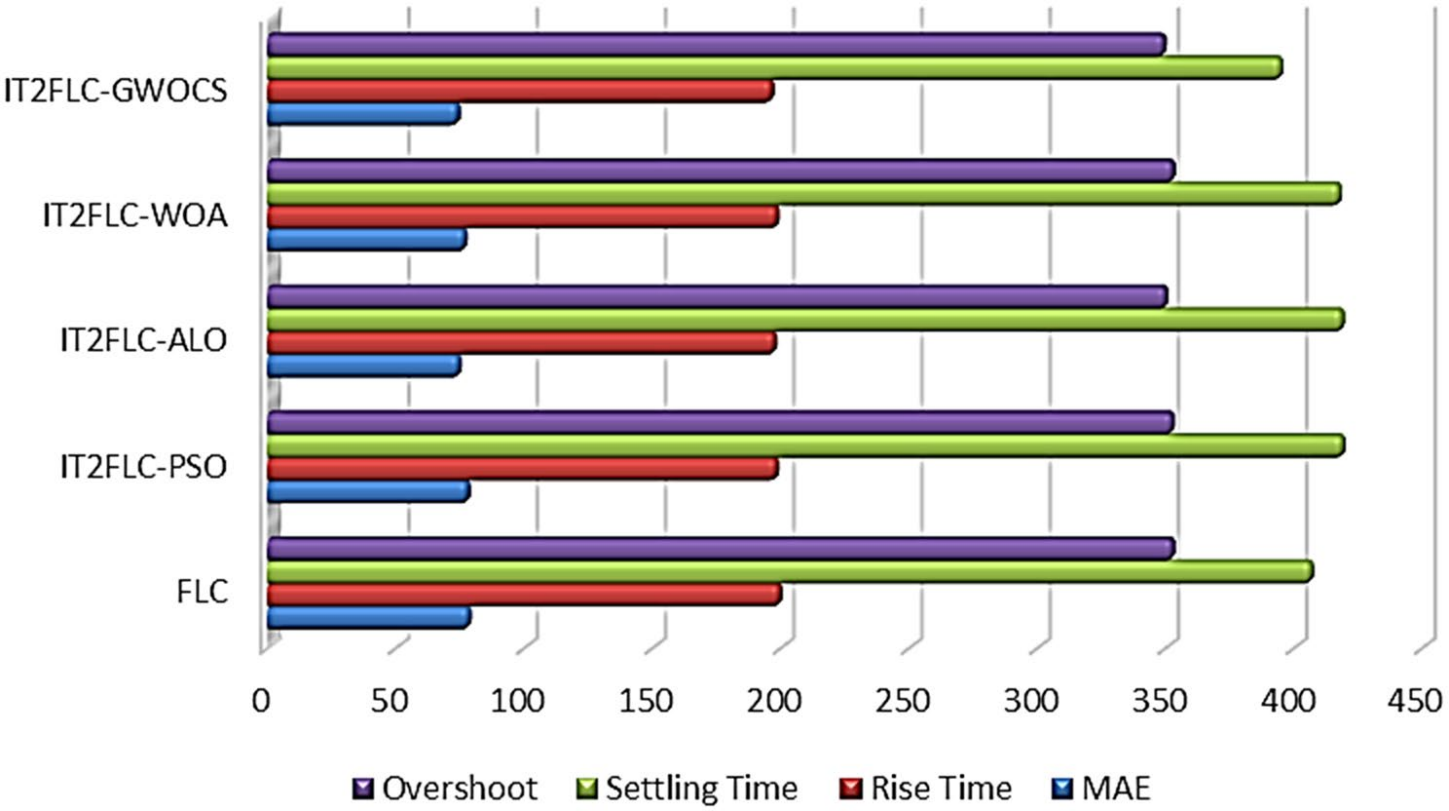
Graph of the Comparison between GWOCS, ALO, PSO, WOA and FLC

We can infer from Figs. 29, 30 and Table 4 that IT2FLC using GWOCS appeared superior to others. It is the least in the MAE, Rise Time, Settling Time, and Percentage Overshoot. So IT2FLC using GWOCS was chosen to be appropriate for a more realistic and different scenarios of T1DPs.

## Conclusion

In this study an effective control approach utilizes IT2FLC with the conjunction of hybrid grey wolf optimizer-cuckoo search (GWOCS) to regulate BGL for T1DP was proposed, the nonlinear EBMM was used to describe the behavior of glucose-insulin physiological system for T1DP. Superiority of IT2FLC in minimizing the effect of uncertainties in the system depends primarily on the best choice of FOU parameters of IT2FLC, so GWOCS was used to optimally tune and select the best FOU that improves the performance of the system. The proposed control approach infused an appropriate amount of exogenous insulin into the bloodstream of T1DP based only on BGL measurements without the need to measure other status variables of remote insulin and plasma insulin level. The controller output of the proposed control method was always non-negative, no excessive controller or algorithm were needed to infuse glucagon or glucose to avoid hypoglycemia and this significantly enhances the simplicity of the design and can reduce complexity and cost.

The effectiveness and the performance of the proposed method were evaluated under six difficult and different simulation scenarios that a T1DP is likely to face in his daily life.

Simulation results indicated that the proposed control approach effectively regulated and stabilized BGL within the standard safe range between 70 and 180 mg/dl even the presence of parametric uncertainty of the model, different meals disturbance and random initial condition of state variables of EBMM. That is in the critical and different scenarios that a T1DP is likely to face in his daily life, the risks of hyperglycemia and hypoglycemia have been avoided.

Stability analysis for the system was performed using lyapunov function and simulation scenarios also proved that the system operates within the stability region of nonlinear system. Consequently the proposed control approach can significantly save life and reduce the chance of death for T1DPs.

One limitations of the proposed control approach is that it takes a fairly long time to obtain an optimal solution due to type-reduction process associated with IT2FLC which is iterative and computationally intensive, however in this work EIASC was utilized to perform type-reduction which can save more 50% computational cost over ordinary KM algorithm.

Implementation of the proposed control approach practically and linked it to a network of medical devices to share real time measurements of BGL may be considered the focus of research in the future.
